# Genome‐Wide Development and Characterization of Microsatellite Markers in the Great Web‐Spinning Sawfly *Acantholyda posticalis*

**DOI:** 10.1002/ece3.70500

**Published:** 2024-11-04

**Authors:** Mengfei Liu, Xiaoyi Wang, Hongbin Wang, Guohong Li, Mingyang Pei, Gege Liu, Mei Wang

**Affiliations:** ^1^ Key Laboratory of Biodiversity Conservation of National Forestry and Grassland Administration Ecology and Nature Conservation Institute, Chinese Academy of Forestry Beijing China; ^2^ Chaoyang Natural Resources Affairs Service Center Liaoning China; ^3^ State‐Owned Lingbao City Chuankou Forest Farm Henan China

**Keywords:** cross‐amplification, genetic differentiation, Hymenoptera, microsatellite markers, population genetics

## Abstract

The great web‐spinning sawfly *Acantholyda posticalis* is notorious for damaging *Pinus* forests across the Palearctic region. At present, uncertainties persist regarding its intraspecies variation and presumed subspecies. To use as tools for future studies, herein we developed genome‐wide microsatellite markers for *A. posticalis*. Through searching, rigorous manual screening, and amplification trial, 56 microsatellite markers were obtained from the genome sequences. We characterized these markers across two populations from Shandong province (SD) and Heilongjiang province (HLJ) in China, and carried out cross‐amplification in three related species. Out of the 56 markers tested, 10, 31, and 15 were categorized into high, moderate, and low polymorphic levels, respectively, based on their polymorphic information content (PIC) values. Meanwhile, 28, 19, and 4 microsatellite loci were successfully cross‐amplified in *Cephalcia yanqingensis*, *C. chuxiongica*, and *C. infumata*, respectively, which could serve as potential molecular markers for their further studies. STRUCTURE and PCoA analyses revealed two distinct clusters corresponding to SD and HLJ, respectively, indicating a high resolution of these markers. Therefore，the 56 microsatellite markers identified here have the potential to serve as efficient tools for unraveling intraspecies variation and evolutionary history of *A. posticalis*.

## Graphical abstract

*Acantholyda posticalis* is notorious for causing serious damage to *Pinus* forests across the Palearctic region. Through genome‐wide searching, manual screening, and amplification trial, 56 microsatellite markers were developed for *A. posticalis*. Our study is the first to develop SSR markers for insects in family Pamphiliidae, representing an initial step toward revealing the population history of *A. posticalis* and improving understanding for future effective pest‐management strategies.

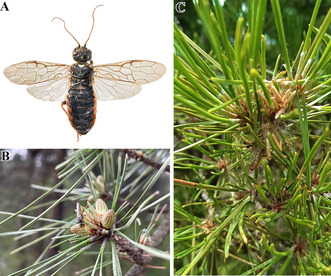

## 1 Introduction

Microsatellites (SSRs) are highly effective molecular markers in population genetics, owing to their high mutation rate, co‐dominant inheritance, abundance, multi‐allelic character, and high information content (Selkoe and Toonen 2006; Guichoux et al. 2011). Traditionally, the development of SSR markers was expensive and time‐consuming, such as expressed sequence tag library enrichment and gene cloning (Zane, Bargelloni, and Patarnello 2002; Squirrell et al. 2003). With rapid progress in sequencing and bioinformatics, it was relatively labor‐saving and cost‐effective to obtain SSR markers of high genome‐wide coverage (Taheri et al. 2018). Single nucleotide polymorphisms (SNPs) have attracted lots of attention recently. However, especially for species without a reference genome, SNP development usually shows higher costs and requires a greater amount of DNA per sample (Grover and Sharma 2016).

**FIGURE 1:**
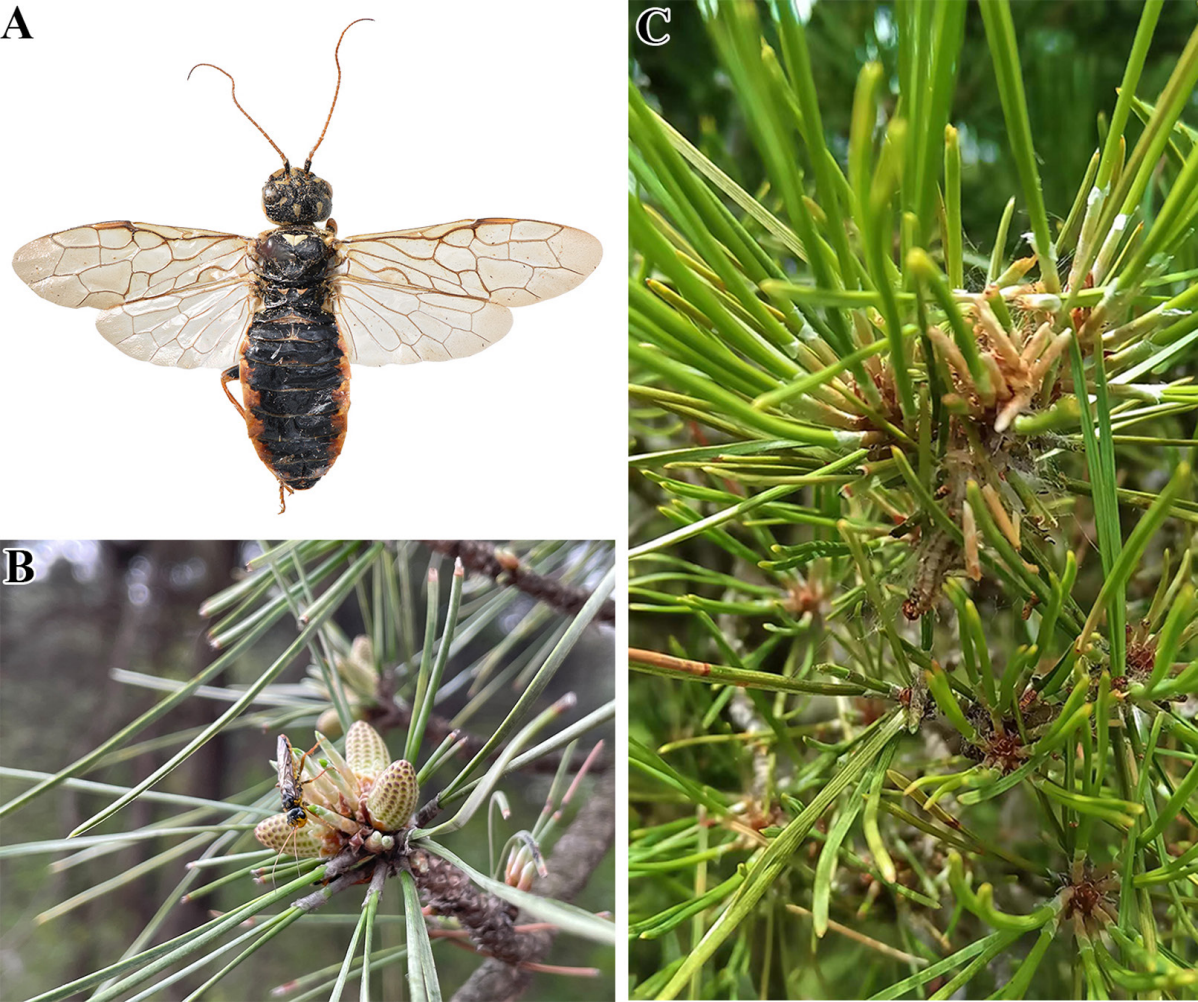
Specimen and ecological picture of *Acatholyda posticalis*. (A) A female adult collected from Shandong province, China in 2022. (B) Newly emerged adult on a *Pinus tabuliformis* tree in Shanxi, China, 2023. (C) Larva feeding with pine needles and symptoms of damage in Ningxia, China. Photo of larva courtesy of Chao Liu (Luoshan National Nature Reserve, Ningxia).

*Acantholyda posticalis* Matsumura (1912) (Hymenoptera: Symphyta: Pamphiliidae), is a forestry pest inflicting severe damage to *Pinus* forests and widely distributed in the Palearctic region (Figure 1) (Kolomyietz 1967; Pschorn‐Walcher 1982; Shinohara and Byun 1996; Xiao 2002). At fourth larval instar stage, the larvae move to the soil where they stay for overwintering or diapause for 1–3 years, or even up to 7 years before they emerge (Kolomyietz 1967; Voolma, Pilt, and Õunap 2009; Ghimire et al. 2013). The highly variable larval stage has made its outbreaks unpredictable (Ghimire et al. 2013). In Shangluo, China, since the first mass outbreak in 2002, over 38,000 ha of *Pinus tabuliformis* plantations had been defoliated (Duan and Meng 2020).

As a notorious web‐spinner, the variations linked with geographical location in *A. posticalis* have been of constant concern, such as their morphology and host preference (Shinohara 2000; Voolma et al. 2016). In Europe and Siberia, Scots pine (*Pinus sylvestris*) is the dominant host (Voolma, Pilt, and Õunap 2009; Sokolov 2014; Voolma et al. 2016; Mussayeva et al. 2019), while in Japan and Korea, it is the Japanese red pine (*Pinus densiflora*), and Chinese red pine (*Pinus tabuliformis*) in China (Shinohara 2000; Xiao 2002). For morphological variations, Shinohara (2000) identified three subspecies correlated with specific distributions: the nominotypical *A. posticalis posticalis* from Japan, *A. posticalis koreana* from Korea, and *A. posticalis pinivora* from Europe, Siberia, and China. However, it needs further validation due to the limited samples and some questionable specimens, such as those from China, where only two specimens are available and do not fully match the characteristics of the identified subspecies (*A. posticalis posticalis*).

Deciphering population evolutionary history and phylogeography can facilitate the development of effective suppression protocols and sustainable management strategies for insect pests (Guillemaud et al. 2011; Meng et al. 2018). Given the economic importance and the limited knowledge in intraspecies variations, it is imperative to investigate the population genetics and dynamics of *A. posticalis* with highly‐resolving molecular markers. Unfortunately, existing genetic resources are scarce, with less than 200 entries in the public database (NCBI 2024) under “*Acantholyda*” as of 1th April 2024 (https://www.ncbi.nlm.nih.gov/nuccore/?term=Acantholyda). A limited number of SSRs studies had been conducted on sawflies (Hartel, Frederick, and Shanower 2003; Cook et al. 2011; Caron et al. 2013; Bittner et al. 2017; Kulanek, Blank, and Kramp 2019), while none was focused on pamphiliids.

Herein, to address this gap and pave the way for future investigations into the population history of *A. posticalis*, we developed genomic SSR markers with multiple factors taken into account and estimated the frequency and distribution of SSRs within the *A. posticalis* genome. To assess the cross‐species applicability of these markers, cross‐amplifications were conducted in three related pamphiliids from a sister genus, which are also forestry pests, including *Cephalcia yanqingensis*, *C. infumata,* and *C. chuxiongnica*. Our study is the first to develop SSR markers for insects in family Pamphiliidae, representing an initial step toward revealing the population history of *A. posticalis* and imrproving understanding for future effective pest management strategies.

## 2 Materials and Methods

### 2.1 Sample Collection and DNA Extraction

**FIGURE 2:**
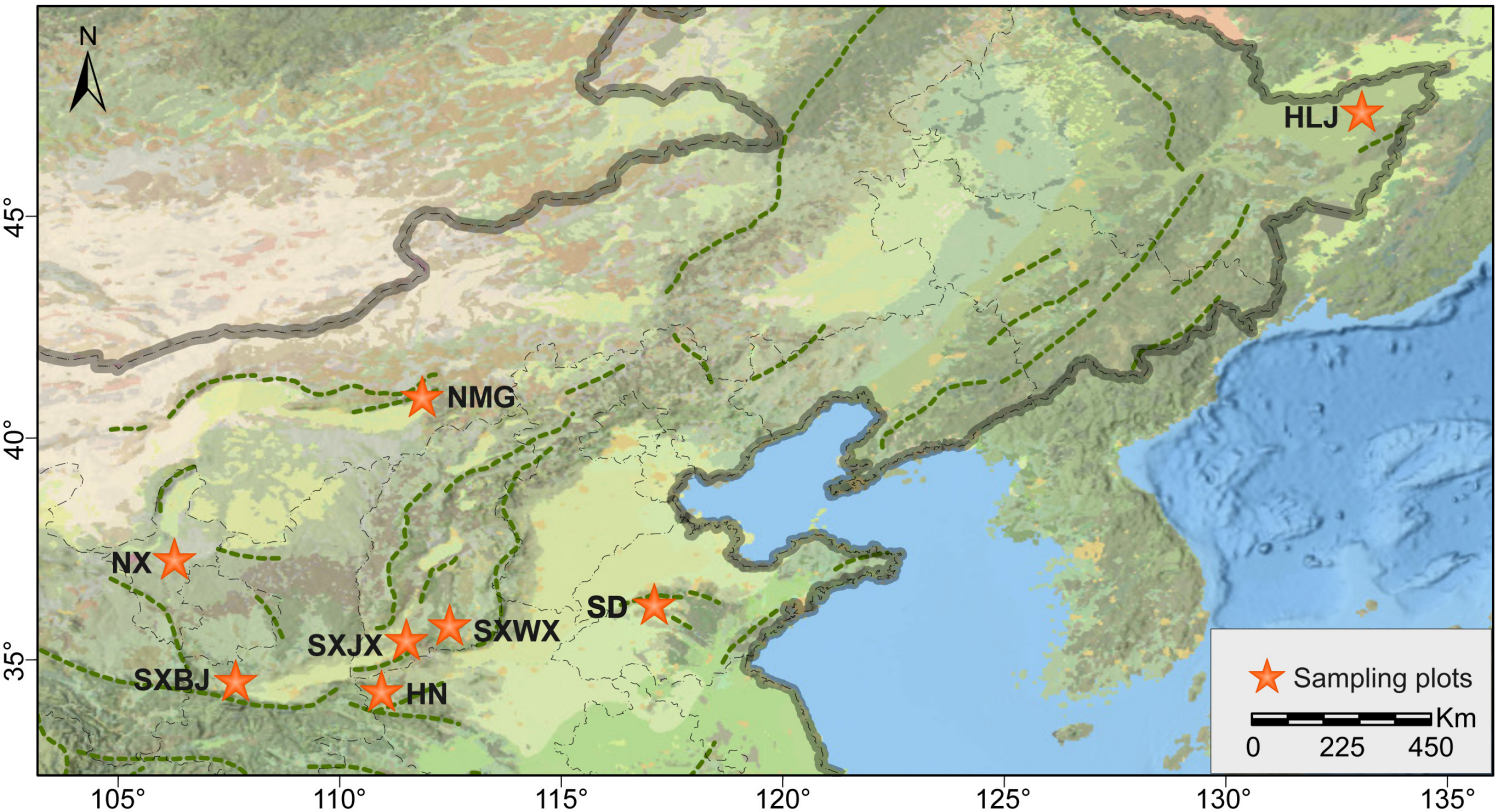
Sampling sites of *Acantholyda posticalis*. The map is based on the standard map of GS (2020) 4619 downloaded from the standard map service website of the National Bureau of Surveying, Mapping, and Geoinformation, and the base map is not modified.

All samples used in the study were collected in infested pine forests during 2022–2023 (Figures 1 and 2; Table S1) and preserved in absolute ethanol at −20°C. Among the specimens, a female adult from Luoshan, Ningxia Province, China was used for genomic sequencing. Eight individuals from different collection sites were applied for initial test of primers. A total of 48 samples from two regions(SD and HLJ)were used to validate the effectiveness of the polymorphic primers at the population level. Total DNA was extracted from thorax using the DNeasy Blood and Tissue Kits (QIAGEN, Hilden, Denmark) in accordance with the manufacturer's instructions. DNA concentration of each sample was measured with NanoDrop One (Thermo Fisher Scientific, Waltham, MA, USA) and then numbered and stored in a freezer at −20°C. Voucher specimens were deposited at the Natural History Museum of Ecology and Nature Conservation Institute, Chinese Academy of Forestry, Beijing.

### 2.2 Library Construction, Genome Sequencing, and Assembly

Genomic DNA of a female adult was taken as input material and a 350 bp paired‐end library was constructed according to the manufacturer's instructions (Illumina): DNA random fragmentation by sonication, end‐polishing of the DNA fragments, poly‐A tailing and ligation with the full‐length adapters, PCR amplification, and purification of PCR products (AMPure XP bead system). Then, the DNA library was sequenced on Illumina Novaseq 6000 platform (Illumina, Inc., San Diego, CA, USA) by Beijing Berry Genomics Co. Ltd. 150 bp paired‐end raw data was generated and quality evaluation was performed through FastQC v0.11.5 (Andrews 2010) prior to assembly. We used SOAPdenovo2 (Luo et al. 2012) to assemble the genomic sequences. Genome size, heterozygosity, and duplication rate were estimated by using Jellyfish v2.2.6 and GenomeScope v2.0 with a K‐mer method (Marçais and Kingsford 2011) and the completeness of the assembled genome was estimated by BUSCO v3 (Simão et al. 2015). The assembled contigs were utilized to identify SSRs subsequently.

### 2.3 Microsatellite Survey

Krait (https://github.com/lmdu/krait) is an ultrafast tool for genome‐wide survey of SSRs (Du et al. 2018). All SSRs were scanned from the assembled genome by Krait with a minimum of 12, 7, 6, 6, 5 and 5 repeats to identify the mono‐, di‐, tri‐, tetra‐, penta‐ and hexanucleotide motifs respectively in perfect SSR search. To simplify the classification, we standardized the circular permutation and reverse complement repeats as the same motif, such as (ATG)_n_, (TGA)_n_, (GAT)_n_, (CAT)_n_, (ATC)_n_, and (TCA)_n_, were considered as the same type. The number of SSRs, as well as the average length (bp), relative abundance (loci/Mb), relative density (Kb/Mb) were analyzed.

### 2.4 Primer Design and Selection

The design and selection of SSR primers were conducted in the QDD v3.1 (Meglécz et al. 2014). The parameters and criteria were as follows: (1) tri‐, tetra‐, penta‐ and hexanucleotide motifs were at least 7, 7, 6, and 6 repeats respectively; (2) the length of PCR products ranged from 90 to 400 bp; (3) the primers need to between 18 bp and 24 bp with the optimal length of 20 bp; (4) the annealing temperature had to between 58°C and 63°C with the optimal temperature at 60°C, and the difference between pairwise primers had to be lower than 4°C. The remaining parameters were at default.

Compared to primers designed based on unique singletons, those derived from consensus sequences are more prone to genotype failure (Meglécz et al. 2014). Hence, to obtain more available primers, further manual filter was conducted with a strict line as follows: (1) the dinucleotide motifs were excluded; (2) the primers from consensus sequences were ignored; (3) only primer pairs with “A” strategy in QDD v3.1 were taken into consideration to avoid homopolymers and compound and multiple SSRs in the amplicon; (4) the minimum distance between the 3′ end of primer pairs and the target region needed to be longer than 10 bp; (5) avoid motifs that can form hairpins [e.g., (AT)_n_]; and (6) only one target region was considered in a contig, and only one primer pair was retained in a target region. After screening, we synthesized the candidate primer pairs for validation (Sangon Biotech, Shanghai, China).

### 2.5 Amplification and Validation of Microsatellite Loci

There were initial and further tests for the effectiveness of candidate SSR markers based on individuals and populations respectively.

First, 188 primer pairs (Table S2) were amplified in eight individuals from different collection sites mentioned above. In order to improve efficiency and lower the cost of capillary electrophoresis, the 5′ end of the forward primers were tagged with PC tail (Primer tail C, 5′‐CAGGACCAGGCTACCGTG‐3′) (Blacket et al. 2012). Additionally, the corresponding universal primers labeled with fluorescence (FAM and HEX) were also synthesized. PCR amplification was conducted in a 20 μL reaction mixture containing 10 μL 2xFlash PCR MasterMix (CWBIO, Jiangsu), 0.16 μL forward primer (modified by PC tail), 0.32 μL reverse primer, 0.64 μL universal primer, 1 μL template DNA and 7.88 μL ddH_2_O. The amplification program was as follows: an initial denaturation at 95°C for 5 min, followed by 35 cycles of denaturation at 95°C for 30 s, annealing at 59°C for 40 s and extension at 72°C for 40 s, with a final 15‐min extension at 72°C. PCR products were visualized by agarose gel (1.5%) electrophoresis. Only when the primers amplified successfully in eight individuals and produced clear and distinct bands, would the products be analyzed in an ABI 3730XL DNA Analyzer (Applied Biosystems, Foster, CA, USA) with the GeneScan 500 LIZ size standard (Applied Biosystems). The results were performed in GeneMaker V2.2.0. Through genotyping, we discarded the loci without polymorphism or showing more than two peaks in one individual.

Second, the remaining primers (Table S3) were validated in two populations (SD and HLJ). All of the products were loaded onto ABI 3730xL DNA Analyzer (Applied Biosystems, Foster, CA, USA) for capillary electrophoresis. The amplification mixture, PCR program, electrophoresis, and genotyping methods followed the above‐mentioned steps.

### 2.6 Polymorphism and Population Structure Detection

Null allele frequencies (*r*) were checked using FreeNA (Chapuis and Estoup 2007) with the EM algorithm (10,000 bootstraps) (Dempster, Laird, and Rubin 1977). Deviation from Hardy–Weinberg equilibrium (HWE) at each locus/population and linkage disequilibrium (LD) in pairwise loci were analyzed through GenePop 4.8.3 (Rousset 2008). Critical significance levels were adjusted for multiple comparisons using Bonferroni corrections when testing HWE and linkage disequilibrium. Polymorphism for each locus was described with multiple genetic diversity parameters. Observed heterozygosity (*H*_O_), expected heterozygosity (*H*_E_), and polymorphism information content (PIC) were calculated in the Excel Microsatellite Toolkit (Trinity College Dublin, Ireland). Number of alleles (*N*_A_), allelic richness (*A*_R_), and inbreeding coefficient (*F*_IS_) were estimated following Weir and Cockerham (1984) using FSTAT 2.9.3 (Goudet 2002).

Measure of genetic differentiation was assessed with Wright's *F*_ST_ (fixation index) (Wright 1978). Here, we calculated *F*_ST_ with and without excluding null alleles (ENA) method in FreeNA (Chapuis and Estoup 2007), testing for the presence of null alleles in the populations and evaluating the impact of null alleles on the analysis of population differentiation. The analysis of molecular variance (AMOVA) was performed by Arlequin 3.5.2 (Excoffier and Lischer 2010). AMOVA was performed to assess the genetic variance partitioned into two levels (among populations and within populations).

There were two approaches utilized to characterize the patterns of population admixture. First, based on co‐dominant genotypic distance among pairs of all individuals and populations, principal coordinate analysis (PCoA) was conducted in GenAlEx 6.5 (Peakall and Smouse 2012). For the second approach, a Bayesian model‐based cluster analysis was implemented in STRUCTURE 2.3.4 (Pritchard, Stephens, and Donnelly 2000) under an admixture model with correlated allele frequencies and no prior geographic information. Ten independent runs were performed for *K* = 1–4, with a burn‐in period of 20,000 iterations, followed by 120,000 Markov Chain Monte Carlo iterations. The optimal *K* value was inferred from the ΔK method (Evanno, Regnaut, and Goudet 2005) in STRUCTURE HARVESTER (Earl and Vonholdt 2012). The membership coefficient matrices (*Q*‐matrices) of the replicated runs at optimal *K* were combined by CLUMPP 1.1.2 (Jakobsson and Rosenberg 2007) under the Greedy algorithm, and the visualization was done on DISTRUCT 1.1 (Rosenberg 2004).

### 2.7 Cross‐Species Amplification

SSR markers developed for one species have proven alternative sources for other related species. For cross‐species applicability analysis, we cross‐amplified these newly developed markers in *Cephalcia yanqingensis*, *C. chuxiongica,* and *C. infumata* with four samples per species. Genomic DNA isolation and PCR amplification were performed as described above. The SSR markers producing specific PCR bands in the expected size range were considered potentially applicable to these pamphiliids. The rate of cross‐amplification was measured using the following equation: Amplification rate (%) = amplicons (bands amplified by PCR) × 100/theoretical amplicons (primer number × sample size) (Lee et al. 2015; Raveendar et al. 2015).

## 3 Results

### 3.1 Genome Sequencing and Assembly

**FIGURE 3:**
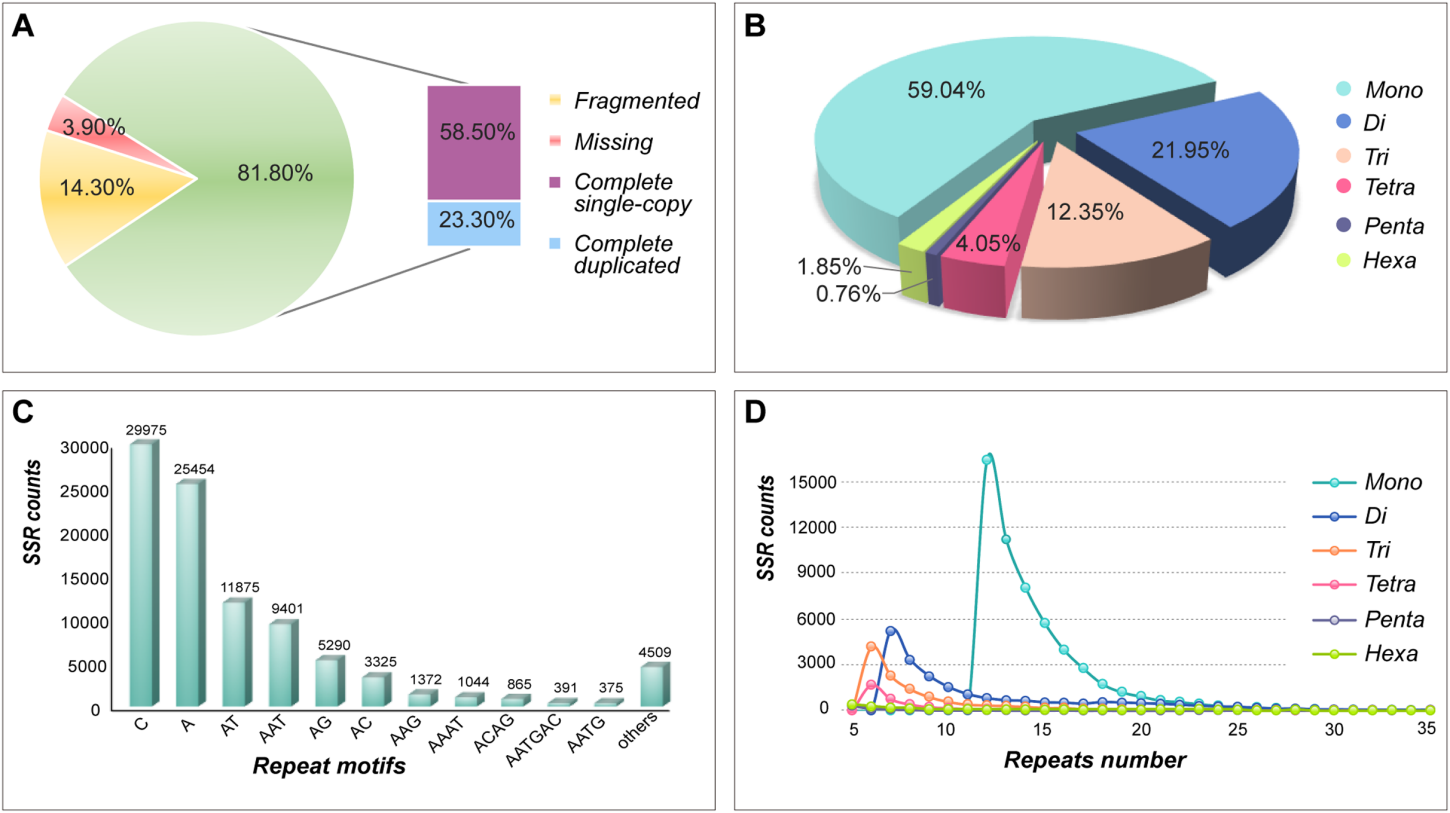
Genome completeness and distribution pattern of microsatellites in *Acantholyda posticalis*. (A) Genome completeness of *A. posticalis* in BUSCO analysis using the Insecta ortholog dataset v.10. (B) Distributions of different microsatellite repeats. Mono, mononucleotide repeats; Di, dinucleotide repeats; Tri, trinucleotide repeats; Tetra, tetranucleotide repeats; Penta, pentanucleotide repeats; Hexa, hexanucleotide repeats. (C) Frequency distribution of microsatellites among different motif types. The “others” category represents summed motifs with counts below 200. (D) Length distribution of microsatellites among different repeats. Mono, mononucleotide repeats; Di, dinucleotide repeats; Tri, trinucleotide repeats; Tetra, tetranucleotide repeats; Penta, pentanucleotide repeats; Hexa, hexanucleotide repeats.

After removing low quality reads, we obtained 40.35 Gb pair‐end sequences with 134,483,802 clean reads. The Q20 and Q30 were 95.88% and 89.87%, respectively, indicating high quality of the genome sequencing. Through K‐mer analysis (*K* = 17) with the clean data (Figure S1), the k‐mer distributions showed double peaks, and the genome of *A. posticalis* was estimated to be 625.47 Mb with the heterozygosity rate of 0.305%, and the duplication rate of 1.59%. At the contig level, we assembled the genome into 691.95 Mb including 90,279 contigs, with the N50 of 12.77 Kb. The genome comprised 37% GC base pairs (Figure S2). In terms of BUSCO annotation with “insecta_odb9” gene set collection, 81.8% of 1367 genes were identified as complete (single‐copied gene: 58.5%, duplicated gene: 23.3%), while 14.3% and 3.9% were fragmented and missing respectively (Figure 3A).

### 3.2 Genome‐Wide Characterization of Microsatellites

A total of 93,876 candidate SSRs were mined from the genome contigs (Table S4). The total relative abundance was 135.67 loci/Mb. The total relative density was 2.73 Kb/Mb (Table S5). The detected SSRs included 55,429 (59.04%) mononucleotide, 20,609 (21.95%) dinucleotide, 11,590 (12.35%) trinucleotide, 3801 (4.05%) tetranucleotide, 713 (0.76%) pentanucleotide, and 1734 (1.85%) hexanucleotide motifs with the searching conditions of SSRs (a minimum of 12, 7, 6, 6, 5, and 5 repeats were used to identify the mono‐, di‐, tri‐, tetra‐, penta‐, and hexanucleotide motifs, respectively) (Figure 3B, Table S5). Overall, a negative correlation emerged between motif length and its abundance; longer motifs tended to be less abundant. Mononucleotide motifs occupied a dominant position. There were 256 different SSR motifs: two mononucleotides, four dinucleotides, ten trinucleotides, 28 tetranucleotides, 64 pentanucleotides and 148 hexanucleotides (Table S6). In decreasing order, the most abundant motifs were (C)n, (A)n, (AT)n, (AAT)n, (AG)n, (AC)n, (AAG)n, (AAAT)n, and (ACAG)n, and (AATGAC)n with the proportion of 94.77% in all identified SSRs (Figure 3C).

Of the 93,876 genomic SSRs, the repetitive number ranged from 5 to 135 and mainly distributed in 5–21, and there were 5190 (5.54%) SSRs with their repeats over 21 (Figure 3D). The repeats of mono‐, di‐, tri‐, tetra‐, penta‐, hexanucleotide mainly distributed in 12–21, 7–12, 6–10, 6–10, 5–8, and 5–8, respectively (Figure 3D, Figure S3). Besides, the average length of different motifs ranged from 14 to 67 bp while the relative abundance and density ranged from 1.03–80.11 loci/Mb and 40.3–1181.63 bp/Mb, respectively (Table S5).

### 3.3 Development and Polymorphism of Microsatellite Loci

Based on the genome sequences, 468,446 primer pairs were designed by QDD3 (Table S7). After stringent manual screening, 188 primer pairs were retained for the initial validation (Table S2). There were 109 loci successfully amplified in all eight individuals, but 25 were rejected due to unclear and faint bands or non‐specific amplification on agarose gel electrophoresis. Through capillary electrophoresis, 69 loci showed polymorphism while 15 were monomorphic.

To generate a reliable genotype dataset, first we took HWE and LD test and estimated the frequency of null alleles. A heterozygote deficit was detected in 16 of the 138 locus‐population pairs, while two pairs showed heterozygote excess after Bonferroni correction (*p* < 0.00036) (Table S8). A total of 17 loci showed that the hypothesis of Hardy–Weinberg equilibrium (HWE) was rejected (Table S8). No locus was significantly linked in the 2,346 locus‐locus pairs after Bonferroni correction (*p* < 0.00002) (Table S9).

There were two loci with a null allele frequency (*r*) > 0.2 in SD and 13 in HLJ (Table S10), suggesting signs of null alleles at these loci (Dakin and Avise 2004; Chapuis and Estoup 2007). Additionally, 13 of these 15 loci also showed a departure from HWE. The presence of null alleles may account for the deviation from HWE. Therefore, we removed these 13 loci and retained the other 56 loci for subsequent analyses (Table S3, Table 1).

**TABLE 1:** Polymorphic parameters of 56 microsatellite markers validated in two *Acantholyda posticalis* populations.

Locus/population	*N*_A_	*A*_R_	*H*_O_	*H*_E_	PIC	*F*_IS_
S1*	2	2.000	0.000	0.505	0.375	—
S2	1	1.000	0.000	0.000	0.000	—
S4*	2	2.000	0.000	0.505	0.375	—
S5	2	1.957	0.063	0.100	0.094	0.349
S10*	2	2.000	0.021	0.503	0.374	0.657
S11*	3	2.989	0.104	0.568	0.466	0.184
S16	1	1.000	0.000	0.000	0.000	—
S18*	2	2.000	0.000	0.505	0.375	—
S20*	2	2.000	0.000	0.505	0.375	—
S21	1	1.000	0.000	0.000	0.000	—
S24*	2	2.000	0.000	0.505	0.375	—
S25	2	1.709	0.000	0.041	0.040	1.000
S27	2	1.458	0.021	0.021	0.020	0.000
S28	2	1.458	0.021	0.021	0.020	0.000
S29*	4	3.936	0.417	0.524	0.465	−0.245
S30**	5	4.802	0.438	0.603	0.553	−0.193
S39**	5	4.789	0.152	0.636	0.564	0.447
S42*	3	2.458	0.021	0.516	0.390	0.000
S47	2	2.000	0.063	0.266	0.229	0.720
S50*	3	2.468	0.043	0.516	0.391	−0.011
S55**	7	6.535	0.208	0.707	0.665	0.496
S64*	2	2.000	0.000	0.505	0.375	—
S76	2	1.845	0.063	0.061	0.059	−0.046
S90	1	1.000	0.000	0.000	0.000	—
S92*	5	4.261	0.104	0.587	0.495	0.377
S94*	3	2.999	0.128	0.588	0.496	0.254
S98	1	1.000	0.000	0.000	0.000	—
S99*	2	2.000	0.104	0.426	0.333	0.579
S100*	2	2.000	0.104	0.389	0.311	0.597
S103**	3	3.000	0.083	0.631	0.553	0.676
S107*	2	2.000	0.021	0.505	0.375	0.000
S110*	2	2.000	0.500	0.379	0.305	−1.000
S114*	4	3.416	0.083	0.562	0.458	0.273
S116	1	1.000	0.000	0.000	0.000	—
S117*	2	2.000	0.000	0.505	0.375	—
S125*	2	2.000	0.000	0.505	0.375	—
S129*	3	2.709	0.000	0.525	0.405	1.000
S133**	4	4.000	0.208	0.714	0.653	0.512
S134	2	1.989	0.063	0.137	0.126	0.514
S141*	2	2.000	0.000	0.505	0.375	—
S148**	3	3.000	0.479	0.631	0.554	−0.917
S149*	3	2.919	0.000	0.544	0.432	1.000
S150*	2	2.000	0.000	0.505	0.375	—
S159**	3	2.999	0.149	0.598	0.507	0.195
S160*	3	2.919	0.000	0.544	0.432	1.000
S162**	8	7.306	0.271	0.711	0.678	0.384
S164**	3	3.000	0.125	0.618	0.535	0.455
S176*	2	2.000	0.000	0.505	0.375	—
S185	1	1.000	0.000	0.000	0.000	—
S188**	6	5.086	0.146	0.678	0.617	0.610
S190*	3	2.845	0.021	0.535	0.419	0.657
S202	1	1.000	0.000	0.000	0.000	—
S205*	5	4.222	0.479	0.446	0.398	−0.480
S206*	2	2.000	0.000	0.505	0.375	—
S207*	2	2.000	0.000	0.505	0.375	—
S213*	2	2.000	0.000	0.505	0.375	—
SD	1.661	1.649	0.087	0.133	0.111	0.356
HLJ	1.464	1.457	0.081	0.088	0.075	0.072
Overall	2.661	2.519	0.084	0.409	0.333	0.243

*Note:* “**” and “*” denote loci with high (PIC > 0.5) and moderate (0.25 < PIC < 0.5) polymorphism respectively, and the remaining ones indicate low polymorphism.

Abbreviations: *A*_R_, allelic richness; *F*_IS_, inbreeding coefficient; *H*_E_, expected heterozygosity; *H*_O_, observed heterozygosity; *N*_A_, number of alleles; PIC, polymorphism information content.

The 56 loci were characterized for two natural populations with 24 individuals per population (Table 1). The number of alleles (*N*_A_), allelic richness (*A*_R_), observed heterozygosity (*H*_O_), expected heterozygosity (*H*_E_), polymorphism information content (PIC) and inbreeding coefficients (*F*_IS_) in HLJ samples were all slightly lower than those in the SD population. Both populations had lower *H*_O_ values than *H*_E_ values (SD: 0.087 vs. 0.133; HLJ: 0.081 vs. 0.088) and displayed positive *F*_IS_ values (SD = 0.356, HLJ = 0.072) with an overall value of 0.243.

Of the 56 loci, the numbers of alleles varied from 1 to 8 with an average of 2.66 alleles per locus and allelic richness (*A*_R_) ranged from 1.000 to 7.306 with an average of 2.519 across all samples. The *H*_E_ values ranged from 0.000 to 0.714, with a mean value equal to 0.409. Meanwhile the *H*_O_ values varied from 0.000 to 0.500 and the mean overall value was 0.084. In general, the *H*_O_ values were lower than *H*_E_ values for each locus, except for S76 (0.063 vs. 0.061), S110 (0.500 vs. 0.379) and S205 (0.479 vs. 0.446). The *F*_IS_ ranged from −0.917 to 1.000 in SD, and from −1.000 to 1.000 in HLJ, showing significant variation in inbreeding levels. There were 18 loci in SD and 8 in HLJ with positive *F*_IS_ values. The PIC values ranged from 0.000 to 0.678, with a mean of 0.333, showing a considerable variation in genetic diversity among loci, which suggested that some loci were highly polymorphic while others were less so. According to the PIC value, 10 loci showed high polymorphism (PIC > 0.5), 31 loci showed moderate polymorphism (0.25 < PIC < 0.5), and 15 loci showed low polymorphism (PIC < 0.25) (Botstein et al. 1980).

### 3.4 Performance of Microsatellite Markers and Population Structure

Based on the 56 SSR markers, the corrected and uncorrected *F*_ST_ values were 0.831 and 0.842 respectively, which revealed a very strong genetic differentiation between SD and HLJ. The *F*_ST_ value tended to be slightly lower when corrected with ENA method. The results of AMOVA also suggested that the most genetic variation occurred between populations (84%), while the variation within populations was only 16% (Table 2).

**TABLE 2:** Analysis of molecular variance (AMOVA) based on 56 microsatellite markers in *Acantholyda posticalis* populations.

Source of variation	*df*	Sum of squares	Variance components	Percentage of variation (%)
Among populations	1	793.979	16.47736	84.31
Within populations	94	288.188	3.06582	15.69
Total	95	1082.167	19.54319	100.00

**FIGURE 4:**
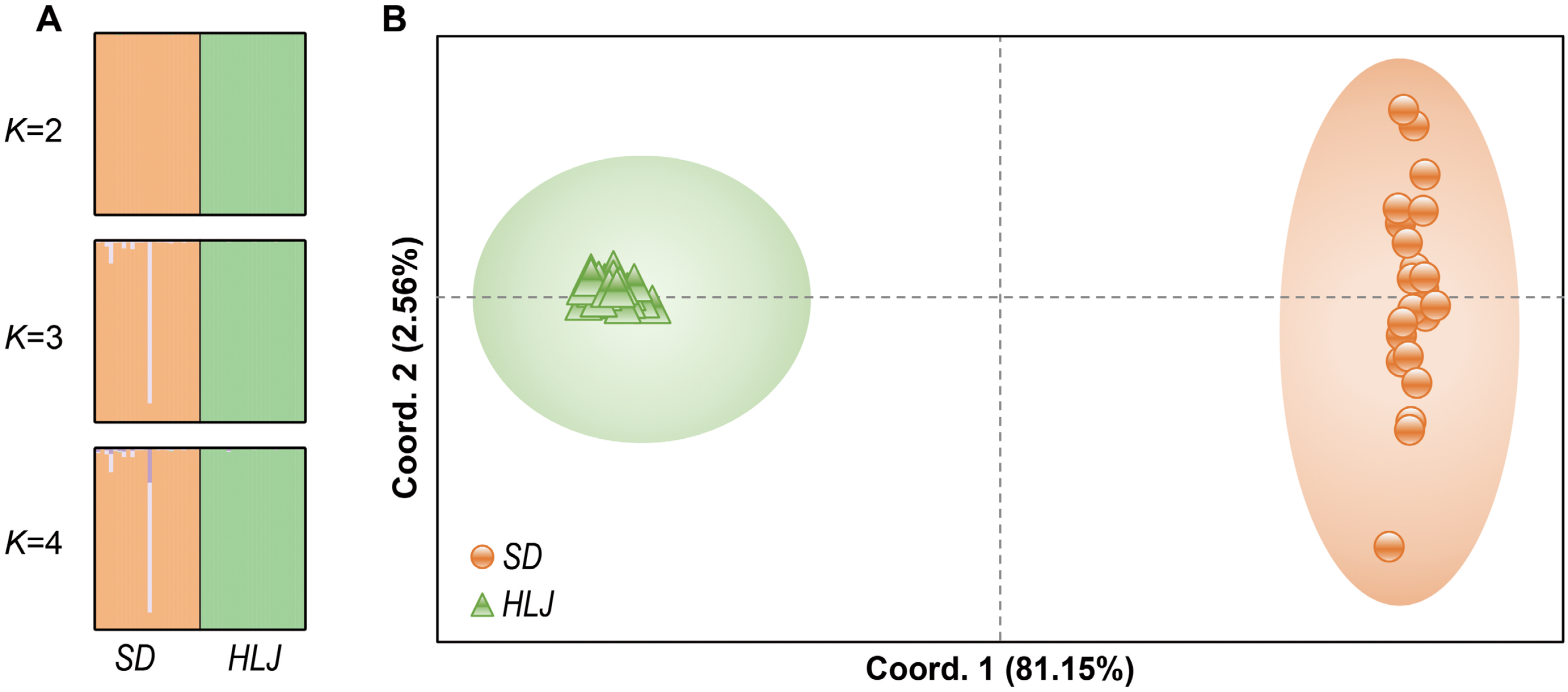
Results of STRUCTURE and PCoA analysis. (A) STRUCTURE clustering for *K* = 2, 3, and 4 based on 56 microsatellite markers. Each vertical bar indicates an individual. Different colors show the identified clusters. The most appropriate clustering was obtained at *K* = 2. (B) PCoA clustering based on 56 microsatellite markers.

Both STRUCTURE (Figure 4A) and PCoA analysis (Figure 4B) based on these SSR markers detected an extremely distinct genetic structure among *A. posticalis* populations. After running STRUCTURE HARVESTER in STRUCTURE analysis, it suggested that the optimal number of genetic clusters were two (best delta*K* = 2), the first cluster comprising SD samples, the second cluster encompassing HLJ samples. The PCoA analysis demonstrated the same pattern that 48 individuals were apparently classified into two groups, and one corresponding to each of the populations. The first principal coordinate and the second principal coordinate accounted for 81.15% and 2.56% of the total variation, respectively, explaining the total genetic variation of the 56 SSR markers.

### 3.5 Cross‐Species Application Evaluation

To assess the cross‐species applicability of the 69 SSR markers of *A. posticalis*, we conducted cross‐amplification in three related sawfly species of the genus *Cephalcia* (Table 3, Table S11). For *C. yanqingensis*, 28 markers were successfully amplified in four individuals. A total of 115 positive amplifications were made with a success rate of 41.67%. For *C. chuxiongica*, 19 markers were successfully amplified in four individuals. A total of 92 positive amplifications were made with a success rate of 33.33%. But for *C. infumata*, there were only four markers were amplified in all individuals. A total of 24 positive amplifications were made with a success rate of 8.70%.

**TABLE 3:** Cross‐species amplification in *Cephalcia yanqingensis*, *C. chuxiongica,* and *C. infumata*.

Species (sample size)	Theoretical amplicons (number)	Amplicons (number)	Transferability (%)
*Cephalcia yanqingensis* (4)	276	115	41.67
*Cephalcia chuxiongica* (4)	276	92	33.33
*Cephalcia infumata* (4)	276	24	8.70

Overall, our SSR markers designed for *A. posticalis* showed the highest amplification efficiency in *C. yanqingensis*, followed by *C. chuxiongica*, while the cross‐amplification in *C. infumata* was worst. Sixteen markers could be amplified in both *C. yanqingensis* and *C. chuxiongica*, and only three of them were successfully amplified in all three species.

## 4 Discussion

### 4.1 Genome Characteristics

Genome survey could provide a preliminary understanding of genomic characteristics of a species. We sequenced random genomic sequences of *A. posticalis*, with a genome size of 625 Mb estimated with K‐mer analysis. This genome size was slightly larger than that of other symphytans, such as *Diprion similis* (Hynmenoptera: Tenthredinoidea) (270.23 Mb) (Davis et al. 2023), *Rhogogaster chlorosoma* (Hynmenoptera: Tenthredinoidea) (255.3 Mb) (Falk and Green 2023) and *Athalia rosae* (Hymenoptera: Athaliidae) (172 Mb) (https://www.ncbi.nlm.nih.gov/datasets/genome/GCF_917208135.1/). The completeness of the assembled genomic sequences was up to 82% (Figure 3A), leading to a good reference for genome‐wide SSR survey. Unlike transcriptome‐derived markers, SSR markers derived from the genome contain abundant neutral markers from non‐coding regions, potentially rendering them less susceptible to selection pressures (Srivastava et al. 2019; Kostro‐Ambroziak et al. 2020). In addition, the assembled genome also provides an important resource for identifying key genes or pathways in *A. posticalis*, such as those involved in environmental adaptation or metabolic regulation (Hüttenhofer and Vogel 2006; Harrow et al. 2009). Although this genome may be sufficient for developing molecular markers or identifying genes, the assembly achieved only with short‐read sequencing and other classical tools is still incomplete (Ji et al. 2011). Whiteford et al. (2005) indicated that while NGS (next‐generation sequencing) reads of 30 bp could generate useful assemblies and recover nearly all genes, genes associated with repetitive elements still failed to be correctly assembled. More different sequencing approaches should be considered for a high‐quality genome (Alkan, Sajjadian, and Eichler 2011), such as long‐read sequencing with PacBio or Nanopore and Hi‐C (high‐throughput chromosome conformation capture) technologies.

### 4.2 Characterization of Microsatellites in *A. posticalis* Genome

As reported, the proportional coverage of different motif types in genomes is variable among species (Meglécz et al. 2012). Of all the genomic SSRs found in *A. posticalis*, mononucleotide motifs were the most abundant category, followed by dinucleotides (Figure 3B; Table S5). In some other Hymenopterans, dinucleotides were the dominant SSR motif in *Platygaster robiniae* (Yang et al. 2021), mononucleotides in *Melipona fasciculata* (Silva et al. 2018) and *Tapinoma indicum* (Lim and Ab Majid 2021), and trinucleotides in *Dolerus aeneus* (Cook et al. 2011). Song et al. (2020) suggested that the variation in SSR distribution patterns among species might be related to their adaptative strategy and evolutionary process. However, the method for mining SSRs can also make a difference to the distribution patterns, such as the sequencing method and SSRs search criterion (Lin et al. 2020; Zhu et al. 2023). As reported in *Tylorrhynchus heterochaetus* (Yang et al. 2023) and *Cyclopterus lumpus* (Maduna et al. 2020) through the genome and transcriptome sequencing, even the same species exhibited different SSR distribution patterns. Besides, some researches set a huge searching length for mononucleotide motifs, leading to a low proportion of it (Song et al. 2017; Wang et al. 2016), or just ignored the mononucleotides (Xia et al. 2018; Shults et al. 2022; Bei et al. 2023), which also contributed to the difference of SSR distribution patterns.

The repeats number of genomic SSRs of *A. posticalis* mainly distributed in 5–21 (Figure 3D). Notably, there was a sharp decline in the counts of SSRs as the repeats number increased, which was consistent with previous studies (Bai et al. 2010; Wang et al. 2016; Liu et al. 2019; Li et al. 2021). It may be linked with microsatellite instability, as instability increases with the length of the repeat (Wierdl, Dominska, and Petes 1997; Lujan, Clark, and Kunkel 2015).

### 4.3 Polymorphism of Microsatellite Markers and Population Genetic Diversity

Through quality checking of the genotype dataset, there was no locus significantly linked between any pair of loci across populations, but there were 17 loci involved in the deviation from HWE, of which 15 loci exhibited a heterozygous deficiency (Table S8). The deviation of the 15 loci from HWE might be correlated to the low *H*_O_, which are much lower than *H*_E_ in these loci. Notably, 13 of them presented high null allele frequencies. The low values of *H*_O_ might be caused by the presence of null alleles, of which the null allele frequencies for the 13 loci are slightly higher than those for the remaining loci. Null alleles have been frequently reported in population genetic studies (Dakin and Avise 2004; Rico et al. 2017; Gstöttenmayer et al. 2023), usually resulting from variations in flanking region sequence (point mutation, insertion or deletion), which prevent primer binding to the template sequence and hinder PCR amplification (Callen et al. 1993). Null alleles thus correspond to genotypes in homozygous form and contribute to heterozygous deficiency (Chapuis and Estoup 2007). In this way, the presence of null alleles may be the major contributor to the deviation from HWE at these loci (Douglas and Trudy 1995; Hedrick 2005; Chapuis and Estoup 2007; Song et al. 2017; Shults et al. 2022). To generate more reliable results, we removed the 13 loci in subsequent population genetic structure and diversity analyses.

The ability of polymorphism detection is crucial for selecting molecular markers. In our study, PIC values varied greatly among loci. There were 10 loci with values greater than 0.5, 31 loci with values between 0.25 and 0.5, and 15 loci with values lower than 0.25. Low PIC values are associated with low or high loci frequencies (Table 1) (Grativol et al. 2010; Serrote et al. 2020). According to Grativol et al. (2010), alleles with high frequency are predominant among individuals, often displaying a tendency toward monomorphism, consequently yielding low PIC values. Likewise, less frequent alleles also tend toward monomorphism due to the prevalence of absent bands, further contributing to lower PIC values (Smith et al. 1997; Grativol et al. 2010). For this reason, Serrote et al. (2020) recommend markers with PIC values above 0.5 rather than those below 0.25. Accordingly, for future studies we recommend to choose, out of the set of 56 SSR markers, the 10 that showed high polymorphism, or to employ a combination of highly and moderately polymorphic markers.

Apart from null alleles, inbreeding within a population may be also responsible for the departure from HWE (Charlesworth 2003). Inbreeding coefficient (*F*_IS_) quantifies the proportion by which the heterozygosity of an individual is reduced by inbreeding (Wright 1922)，ranging from −1 to 1, where a positive value signifies the presence of consanguineous mating within the population (Weir and Cockerham 1984; Villanueva et al. 2021). In our study, both populations displayed positive *F*_IS_ values, corresponding with lower *H*_O_ values than *H*_E_, which indicated a deficiency of heterozygotes and suggested a high level of genetic relationship among the individuals from the same populations. Such mating reflected the population might have experienced bottleneck effect recently (Lynch, John, and Reinhard 1995). Furthermore, inbreeding can affect individual and population diversity and dynamics (Charlesworth 2003; Kardos et al. 2023). Combined with the overall results of *N*_A_, *A*_R_, both populations showed low level of diversity, which may also be linked with the parthenogenesis in *A. posticalis*. As reported in many other studies, parthenogenesis can result in substantial loss of microsatellite heterozygosity in the offspring (Alavi et al. 2018; Kulanek, Blank, and Kramp 2019).

### 4.4 Resolution of Microsatellite Markers and Intraspecies Variation

*F*_ST_ values greater than 0.25 represent very great genetic differentiation (Wright 1978; Hartl and Clark 1997). The results of the AMOVA and *F*_ST_ analysis confirmed that low levels of diversity were present within populations, while high levels of genetic differentiation were found among populations. Sawflies are known to disperse poorly due to their weak flight capability (in comparison to insects of similar morphology), requirement for high humidity and host‐plant specialization (Benson 1950). It suggested that the geographical distance might restrict gene flow between SD and HLJ, thus promoting population divergence.

In our study, based on the SSR markers newly developed, both STRUCTURE and PCoA analyses revealed two distinct clusters, corresponding to SD and HLJ respectively (Figure 4), implicating that *A. posticalis* from SD and HLJ are genetically highly structured. The set of SSR markers turned to be powerful, presenting high resolution for genetic variation at population level. In numerous studies, SSR markers combined with some important mitochondrial or nuclear genes have been widely used to uncover the genetic structure and evolutionary biology, such as the pink rice borer *Sesamia inferens* (Tang, Lu, and Du 2022), the oriental fruit moth *Grapholita molesta* (Wei et al. 2015), the oriental fire‐bellied toad *Bombina orientalis* (Yu et al. 2021) and the Oriental dobsonfly *Neoneuromus ignobilis* (Lin et al. 2022). Given the morphological variation in *A. posticalis* (Koehler 1954, 1957; Lee 1961; Shinohara 2000), the combined utilization of these SSR markers with mitochondrial or nuclear genes, along with morphological traits, can be foreseeable for forthcoming studies on *A. posticalis*.

### 4.5 Cross‐Species Application

SSR markers have demonstrated utility in related species (Mason 2015). Based on our newly developed SSR markers, we performed cross‐amplification in three web‐spinners. Compared to the previous studies on Hymenoptera, or other insects (Hartel, Frederick, and Shanower 2003; Cook et al. 2011; Caron et al. 2013; Kulanek, Blank, and Kramp 2019), the cross‐amplification efficiency were generally low in our study, viz., 41.67% in *C. yanqingensis*, 33.33% in *C. chuxiongica*, while only 8.70% in *C. infumata*. The cross‐species performance of SSR markers usually depends on the conservation of flanking region (Primmer, Møller, and Ellegren 1996; Barbara et al. 2007; Oddou‐Muratorio et al. 2009). Due to the involvement in gene expressions and biological functions, transcribed sequences are typically more conserved across different taxa than genomic sequences (Siepel et al. 2005). Hence, genome‐derived markers tend to have lower cross‐amplification efficiency compared to transcriptome data or ESTs (Ince, Karaca, and Onus 2010; Ince, Karaca, and Elmasulu 2014; Yan et al. 2017; Xu et al. 2020). Additionally, the conservation of flanking region is closely associated with the evolutionary distance among species (Hartel, Frederick, and Shanower 2003; Xiao et al. 2016; Kpatènon et al. 2020; Thakur et al. 2022; Sun et al. 2024). Most studies chose species in the same genus to evaluate the transferability (Bang et al. 2019; Savadi et al. 2023; Sun et al. 2024), suggesting that our SSR makers may perform better when applied on *Acantholyda* species, such as *A. erythrocephala* in Europe and America.

We successfully amplified 28 markers in *C. yanqingensis* and 19 markers in *C. infumata* (Table S11), which could be applied for their population genetic studies further, while these markers should be used with caution due to the small sample sizes. In addition, SSR primers transferred to closely related species are more likely to display null alleles than those species designed directly, as the changes in flanking region sequence should be more frequent between vs. within species (Flanagan et al. 2002; Oddou‐Muratorio et al. 2009).

## 5 Conclusions

In summary, 56 SSR markers were mined and validated for *A. posticalis* through searching from the genomic sequences, rigorous manual screening, and amplification trial. It was the first attempt to develop SSR markers for insects in family Pamphiliidae. Out of the 56 markers, 10 markers with PIC > 0.5 or a combination of those with PIC > 0.5 and 0.25 < PIC < 0.5 were recommended for further research. Despite the low cross‐amplification efficiency in *Cephalcia* species, those primers successfully amplified still held the potential as molecular sources for *C. yanqingensis*, *C. chuxiongica,* and *C. infumata*. Moreover, based on the genotyping results, inbreeding and parthenogenesis may explain the low intrapopulation diversity, while geographic distance and hence low gene flow may contribute to the high interpopulation diversity and genetic structure. Thus, the markers developed here are anticipated to serve as efficient tools for revealing evolutionary history of *A. posticalis* on a larger scale, predicting potential occurrence and dispersal pattern, thereby facilitating more effective pest management strategies.

## Author Contributions

**Mei Wang:** conceptualization (equal), funding acquisition (equal), project administration (equal), writing – review and editing (lead). **Mengfei Liu:** conceptualization (equal), data curation (equal), formal analysis (equal), investigation (equal), methodology (equal), resources (equal), software (equal), validation (equal), visualization (equal), writing – original draft (equal). **Xiaoyi Wang:** funding acquisition (equal), project administration (equal), supervision (equal), writing – review and editing (equal). **Hongbin Wang:** writing – review and editing (equal). **Guohong Li:** writing – review and editing (equal). **Mingyang Pei:** resources (equal). **Gege Liu:** resources (equal).

## Conflicts of Interest

The authors declare no conflicts of interest.

## Supporting information


Appendix S1.

Appendix S2.

## Data Availability

The datasets generated for this study can be found in the online repositories. The genomic scaffolds of *A. posticalis* were deposited to NCBI under the accessions JBBVMP000000000.
