# Supplementary material for: Genome‐Wide Development and Characterization of Microsatellite Markers in the Great Web‐Spinning Sawfly Acantholyda posticalis
Source: Ecol Evol. 2024 Nov 4;14(11):e70500. doi: 10.1002/ece3.70500 (PMC11534431; doi:10.1002/ece3.70500)
Supplement: Supplementary file 1 — Appendix S1. [file ECE3-14-e70500-s001.docx]

***Supplementary Material***

**TABLES**

- **Table S1.** Sampling information of *Acantholyda posticalis*.

| Sample code | Province | Sampling location | Host | Sampling date | Latitude/Longitude |
| --- | --- | --- | --- | --- | --- |
| SD | Shandong | Tai’an | *Pinus tabuliformis* | 202204 | 36.26 N, 117.10 E |
| HLJ | Heilongjiang | Jiamusi | *Pinus sylvestris* var. *mongolica* | 202205 | 47.38 N, 133.07 E |
| NMG | Inner Mongolia | Hohhot | *Pinus tabuliformis* | 202205 | 40.94 N, 111.86 E |
| NX | Ningxia | Wuzhong | *Pinus tabuliformis* | 202205 | 37.28 N, 106.27 E |
| HN | Henan | Lingbao | *Pinus tabuliformis* | 202205 | 34.30 N, 110.94 E |
| SXWX | Shanxi | Wenxi | *Pinus tabuliformis* | 202205 | 35.27 N, 111.46 E |
| SXJX | Shanxi | Jiangxian | *Pinus tabuliformis* | 202304 | 35.35 N, 111.63 E |
| SXBJ | Shaanxi | Baoji | *Pinus tabuliformis* | 202304 | 34.54 N, 107.65 E |

- **Table S2.** Amplification conditions of 188 primers initially selected and synthesized.

| Locus | Scaffold | Motif | Forward primer 5’=>3’ | Reverse primer 5’=>3’ | Size (bp) | Note |
| --- | --- | --- | --- | --- | --- | --- |
| S1 | contig10250 | (ACGG)_7_ | CTCAGGGAACATTGCTGTGC | TTGCTCCTGTCCGTTAGCC | 95 | polymorphism |
| S2 | contig10307 | (ATC)_7_ | CGCTCGTCTCATAGCCTGAA | TGCAACATTGTGAAGGGAGTAC | 157 | polymorphism |
| S3 | contig10762 | (AAG)_7_ | ACGACCTCTGTATAAACCAGCC | TAAACGCCTGCTCACGTGAT | 199 | low-success-rate |
| S4 | contig11118 | (ACT)_7_ | GTGCTTTCTTTGTCAGGCCG | TGAACCATGTACCTCCAAGCT | 121 | polymorphism |
| S5 | contig11438 | (AACCT)_8_ | ATCGACCGTTTGACAAATATGAGT | CGCAAGCCTTAGGATGTGGA | 201 | polymorphism |
| S6 | contig11447 | (AACCT)_6_ | TCGTTACCCGCCGGATTTAG | CTCTTGAATGTTTGGACTCCGT | 114 | faint bands |
| S7 | contig12020 | (ACT)_9_ | CGAAACCCGGGCATACCTTT | AGTCTGAGCGGGTAACAACC | 173 | low-success-rate |
| S8 | contig12095 | (AAG)_7_ | ACTGTTCTGCTAATGCTATCACAT | GCACATGCACGAAGAGGAAT | 93 | no polymorphism |
| S9 | contig12183 | (AAG)_9_ | TTGGCTTAGGCAACGTGGC | CAAGAAAGCTGCCACAGTCG | 123 | low-success-rate |
| S10 | contig12253 | (ACT)_9_ | ACCTCAGTGCTTCTACCTGG | TCGCGCAGTTTCACGGATAG | 177 | polymorphism |
| S11 | contig12692 | (ACC)_8_ | CCAGGCTCTGCGGATGTT | GGTGATGTACCGAAGTTTGAGA | 147 | polymorphism |
| S13 | contig12729 | (ACAGCG)_6_ | TATTGCGCTGACGACGGAAG | ACGACAACGACAGCGACAA | 240 | non-amplification |
| S14 | contig12761 | (AATAT)_7_ | TCGTTGATTACCCGAAGCCC | CGGACACATTCGGAGAGCTT | 260 | non-specific amplification |
| S15 | contig12882 | (AAAT)_8_ | ACCGCGGAATTTCGAGTGAA | ACGTTCGCTACTAGTCCCTG | 322 | non-specific amplification |
| S16 | contig13346 | (AAC)_8_ | CCTCTGCGAGATCAGCAACA | TCACCTGACAACGGCTTTCT | 116 | polymorphism |
| S17 | contig13382 | (AAG)_7_ | CCCTATGATGGCGAGCGTTA | TCGAAATTTGTTCACTTTCATCGC | 280 | low-success-rate |
| S18 | contig13696 | (ATC)_10_ | CGTCCCTCCTATAACCGTACA | ACCTCGGTTTCACAGCTTCA | 146 | polymorphism |
| S19 | contig14154 | (ACGC)_8_ | ATAGGATCTCAGCAGCGTGC | CAGGGCTCCCGATTCTATGC | 158 | low-success-rate |
| S20 | contig14257 | (AAG)_7_ | GCATCAGACTGCACAAACGC | ACGGAAGATCGAAGCAGACG | 207 | polymorphism |
| S21 | contig14539 | (AAG)_7_ | AATCAACTGGCTGATTGGCG | TCCCTAGATTCGGAGCGATG | 140 | polymorphism |
| S22 | contig15519 | (ACT)_7_ | TCCATTATCGACTCAGCGGC | GCGAACTTCCCGAGAACGAA | 172 | low-success-rate |
| S23 | contig15624 | (ACT)_8_ | CATCTGGGCGATCGGTTCAT | TGCAGTCTGACGTAAATATGACCA | 195 | low-success-rate |
| S24 | contig15737 | (AAG)_7_ | GGCTCAAGTCACGTCGTGTA | GCACTTCAACTGCATGATTGGT | 159 | polymorphism |
| S25 | contig15920 | (AAG)_8_ | AGGCTGATCGTAAACCTCGT | GAGTGGGCGCGAGATAAAGT | 145 | polymorphism |
| S26 | contig16142 | (ACGCTC)_14_ | ACACTCACTCACACCCACAC | GGTGTGAGTGATTGTGAGTGTG | 193 | non-amplification |
| S27 | contig16553 | (AAG)_9_ | AGCGAATAGTCAACTCCAACCT | AGACGTCGAAATCGGTGTATTG | 111 | polymorphism |
| S28 | contig16726 | (AAG)_8_ | TGGCAATGAGTTACGAGATCG | CGAACAAATCTGTGCACCTCC | 225 | polymorphism |
| S29 | contig17544 | (AAG)_9_ | TATGTGCCAAATGCCGAGGT | CACCCACATTTCGGAGAGCT | 297 | polymorphism |
| S30 | contig17623 | (AAG)_8_ | CGCCTCGAATAACGTCAATCC | GCGCGTGGAATGTAAATCCG | 260 | polymorphism |
| S31 | contig18080 | (AAAT)_7_ | AATGCACGATTCGGGAGCAA | GACATTGCACTGCGAGTTCC | 186 | low-success-rate |
| S32 | contig18161 | (AACCT)_6_ | CGGCATAACAAGAGTGCGAG | CCTCGGATCGTCGACCAAAT | 110 | non-specific amplification |
| S33 | contig18627 | (AAG)_7_ | TATAGGCCACCTGTCGTGC | CCTGATACGAGCGTCAGTTGA | 189 | non-specific amplification |
| S34 | contig19108 | (AAC)_7_ | CAAACAGGGAAGGACGGATGA | GGCGGCAACAAATGAAGTCT | 119 | faint bands |
| S35 | contig19652 | (AACCT)_6_ | GCGGACCTTTGACCTCTGAG | GAAGACCTGACAGTGTCCGC | 170 | non-amplification |
| S36 | contig19740 | (AAG)_7_ | AGATCTGTGAGCCGTTCGC | CTCGAGAGTGCGCCTATGTT | 141 | polymorphism |
| S37 | contig2071 | (ATC)_7_ | TTTCCCTCCGAATGGTGCC | TGTCCATTGACACGACAGCG | 293 | low-success-rate |
| S38 | contig20829 | (AAG)_7_ | TGCAACGAATTGGATAAGTGACAC | AACTACCTCGCACCATTTGG | 362 | low-success-rate |
| S39 | contig20829 | (AAG)_7_ | AATAATGGTGTCGGCCAGCT | CCCTCCCAAGCTACACTCTTC | 211 | polymorphism |
| S41 | contig20956 | (AAG)_9_ | TCGTTTCCTTGACAGGTGTCT | GGATGCCTGGTCACGTTACT | 230 | polymorphism |
| S42 | contig21076 | (AAG)_7_ | TGGTTGACACTGGTTTCTTTGC | GTCAAACACGATTCTCGAGACA | 150 | polymorphism |
| S43 | contig2115 | (AATC)_10_ | GTTGTTGGCACAGAAGCAGG | TGGTAGCCGAAACTGGACTG | 149 | no polymorphism |
| S44 | contig21176 | (ACCAGT)_10_ | AGTAGAGCACAGTCAGCTGC | AATGTCGGGCTCACATTGGT | 208 | low-success-rate |
| S47 | contig21247 | (AATG)_7_ | GCCAAGTTATCTGCGCCATC | ATCGACTTCTTCAGACAATCTGTT | 130 | polymorphism |
| S48 | contig21607 | (AGC)_8_ | CTACACAGCAGACAGGAGGG | GTTTGGTCAGAACAGACGGG | 240 | polymorphism |
| S49 | contig21702 | (AAAT)_7_ | TTCGCAATTTCACGCAGAGC | CATTTGTATTTGGCAGACGTTGT | 112 | no polymorphism |
| S50 | contig21837 | (AAAT)_7_ | TACTCGCACTTGACCGGTTT | CCGCCATTCCACCACCTATT | 242 | polymorphism |
| S51 | contig21912 | (ATC)_8_ | CATTCAGCCACTCGGTAGCC | GCACGGACCTTAAGTTGGCA | 95 | low-success-rate |
| S52 | contig22119 | (AACCT)_8_ | TTTCGCATGTCCGTCGAACA | CAAACGCTTCGTCTTGTGGT | 141 | low-success-rate |
| S53 | contig22325 | (AAC)_7_ | CAACGACAACAGCAGAGTGC | TTTGCTCAGGTCGCCTTCAT | 228 | no polymorphism |
| S54 | contig22327 | (ACC)_8_ | GAGGGTGTAAGTATGTCGCCAT | ATGGTCTTACGTGGAGCGAC | 190 | no polymorphism |
| S55 | contig22468 | (AATGAC)_8_ | TGACTGACTTTATGTGGAATCCCT | ACGTCAACAGCGGCTCATTA | 309 | polymorphism |
| S56 | contig22674 | (AATAG)_7_ | TCAGAGTGGCAGATATTTGTTTCA | TCAATGTGGACTATGGTCTTGC | 127 | low-success-rate |
| S57 | contig23074 | (ATC)_7_ | TGGTGGTTCCAGCGTGAAC | ATGTGCAGATCGTGTCAGCA | 194 | low-success-rate |
| S58 | contig23138 | (AAC)_7_ | GAACACCTCGACTCCAACGA | CGGAGTTAAGGTGTCGGTGT | 196 | non-amplification |
| S59 | contig23458 | (AAAT)_7_ | CCTGCGGCACTTCAATGAAA | AAACCACGATCCTCGCCATC | 295 | low-success-rate |
| S60 | contig23818 | (AAG)_7_ | GGTAAACCAAGGGCAAGATGG | CATGCGACATCCTCACGACT | 300 | no polymorphism |
| S61 | contig23934 | (ACAT)_9_ | TGGGAGAGGATATGAAAGTCTTGT | TCAGGCAGAAGTGACAATGAAGA | 186 | low-success-rate |
| S62 | contig24095 | (AACCT)_9_ | TCCTTCAAGGACTTAGCGTTGA | CCTACCGGTTCACACCATCC | 207 | polymorphism |
| S63 | contig24281 | (ACT)_9_ | AAACACCCTGTACAAGGCGC | GGCAACAACCAACTCAAGGG | 143 | polymorphism |
| S64 | contig24429 | (AAAT)_7_ | ATTCCATAATAACGCTTGCCGA | GCTTAGCACCATTTACGGCC | 140 | polymorphism |
| S65 | contig24432 | (AAG)_8_ | CCACAATGCTTCCGCCATTC | GAACTGGGTGATTGGCGAGA | 101 | non-amplification |
| S67 | contig2485 | (ATC)_7_ | ACCTCGAAACATGTGTCTTTCTTG | TAAACGGGATCGGAGGTTGC | 298 | no polymorphism |
| S69 | contig25303 | (AAG)_7_ | AGGCTTGACATGAGACAAGAAGT | GCACAGGAACGAAACGGAAG | 270 | non-specific amplification |
| S70 | contig25304 | (AAAT)_8_ | TCAACCGTGCATCTCTGACA | GGAGCTTACTAAATCCAAACGTGT | 222 | low-success-rate |
| S71 | contig25546 | (AACCT)_8_ | TCGTAGAACATTCGCGCAGA | TATCTGAGAACACCTGCGGC | 205 | non-amplification |
| S72 | contig26031 | (AAG)_7_ | TGAGCATGGTTACATAAAGCGA | TAACTGGCTGATTGGCGCAA | 95 | low-success-rate |
| S73 | contig26044 | (AAG)_9_ | GCACTGCCAGCTTGAATACG | CCGACGATGAATGCTGAAGG | 194 | faint bands |
| S74 | contig26527 | (AAG)_13_ | GTTGGCATTAGCAAGCTGGG | CTACAATCCGGCTGGCTGTT | 160 | polymorphism |
| S75 | contig26562 | (ACCGTG)_12_ | TGACAGTGGTAGTGGCAGTG | TTAACGAACAACCCTCGGCA | 373 | low-success-rate |
| S76 | contig26969 | (AAC)_7_ | AATACGGAACTCAGCGAGGC | TGCACCCGCTATCGAGTAAC | 225 | polymorphism |
| S77 | contig27112 | (AACCT)_8_ | AATAGAAAGGTTTGATGGACAGGG | GCAACTTATGGCGTCATCGG | 190 | low-success-rate |
| S78 | contig27262 | (ACT)_10_ | AGGCCCGTTGATGGTCTAAC | CGTAGATCATTAAATCTTCCCGCC | 161 | low-success-rate |
| S79 | contig27266 | (AACCTC)_9_ | TACTTGAATGAGTGGCCGCA | CTCAGCCCTCAGCCCTGA | 200 | low-success-rate |
| S80 | contig27416 | (ACAT)_16_ | CACAATCGGCTTCATCACGC | AGCCTCCTCCATTCCATGAA | 220 | low-success-rate |
| S81 | contig27530 | (ACG)_7_ | CATAACCGTGCCAAAGCCAC | CGCAGCGGTCGTTCTTATTC | 100 | no polymorphism |
| S82 | contig28207 | (AAC)_8_ | GAGGGTGTTGCATCCTACCC | ATCCGCCATTGTTGCCTAGT | 234 | low-success-rate |
| S83 | contig28330 | (AATGG)_7_ | GCAGAACTGGAGATGTGGCT | CCCTGCTACCAACTACCGAG | 139 | low-success-rate |
| S84 | contig29144 | (ACC)_7_ | CGCAGGAGGTCAAGGACAG | GCGATCGAGCGAGAACTGTA | 357 | no polymorphism |
| S85 | contig29372 | (ATC)_9_ | AACTGTGTTGCGCCTACTGA | GCCAATTAGCTGCAGATGGC | 116 | polymorphism |
| S86 | contig29476 | (AAG)_7_ | GCTGATTGCAGGCTTACGCA | TCTGATCCTCCCTGTAATGGC | 240 | low-success-rate |
| S87 | contig29618 | (AAG)_7_ | GGCCACTAGTCGTCTCCGTA | GTGATCGGAGCCCTCATAGC | 131 | low-success-rate |
| S88 | contig29636 | (AAG)_8_ | ACTTCTGCAGGAGCGAGAAC | TCCTTTATGGCCTCGTGCTG | 296 | low-success-rate |
| S89 | contig29816 | (ACAT)_13_ | TGGAGGGCTGTTTCCTTTGA | TTCACCTGGATCTACGTGTATGT | 304 | low-success-rate |
| S90 | contig3012 | (AAG)_7_ | TCGAATACTCGCTCCAAGCC | CATCAAGCGTCAAACCGAGC | 193 | polymorphism |
| S91 | contig31420 | (ATCC)_7_ | GGCTAATCTCACCCAGGCAT | ACGGTAATCTTCCACGTCCC | 175 | non-amplification |
| S92 | contig31599 | (AACCT)_8_ | CGCGGATGATTAATAGCGCG | TGTTCCGGAGAGTCAAGTCC | 167 | polymorphism |
| S93 | contig31749 | (AAG)_14_ | CGACAATTGACGCTCGAATCT | TACCGAAGCTGTCTTGACCC | 143 | low-success-rate |
| S94 | contig31990 | (AATCG)_6_ | CACAACTCGCGTCATTCGAC | TCGAGATTCGATTCGGCCC | 190 | polymorphism |
| S95 | contig32118 | (AAG)_8_ | CCCACAATGACCAAGCATCC | AGTGGGAGAGGTCCTTTCTGT | 240 | faint bands |
| S96 | contig32307 | (AAG)_7_ | CCAGGAGGGTCAATACGGGA | CACTGGTAGTGCACACCGAG | 198 | non-specific amplification |
| S98 | contig32390 | (AGC)_7_ | TCTTCGTGTTCCTGGTCTGG | AACAGGCTTCGGAGTACAGC | 142 | polymorphism |
| S99 | contig32474 | (ATC)_7_ | ACGTAGGATTCCACAGTGCG | CTATGGACTTGCCTGCACCT | 218 | polymorphism |
| S100 | contig3275 | (ACCT)_8_ | ACCTGGCAAGAGTCTGAAAGT | ACAGGCGTCACTTGATCACT | 285 | polymorphism |
| S101 | contig33110 | (AACCT)_7_ | AAACTGTAACTGAACCGTGAAGT | ACTGTGCCGAGAAATTGTTCC | 269 | no polymorphism |
| S102 | contig33908 | (ATC)_7_ | TGCAGATTCAGGGACACGAC | TTTCTCCTCGCACCGATCAC | 153 | low-success-rate |
| S103 | contig3396 | (AAC)_7_ | GGTGTCAGCACGACTCAGAA | CCGGTCCTCGCTTTGTAGTT | 196 | polymorphism |
| S104 | contig33978 | (AAG)_8_ | ATCAAATTGCGTGGCTGCTC | ATTCTGTTGGGTCACGGAGC | 250 | low-success-rate |
| S105 | contig34571 | (AAC)7 | CTGAACAACATTCGCGAGGC | CGCGTAGATGAAGGTCACCA | 234 | non-amplification |
| S106 | contig34708 | (AAG)_10_ | TGAAGGTACTGAACGCCACG | GCGCGTGGGTGACTATTGTA | 179 | low-success-rate |
| S107 | contig34763 | (AAG)_10_ | TGCACTATCTTCAATCTCGGACA | GAAGGAGGAACAGTGGCGTT | 222 | polymorphism |
| S108 | contig35137 | (ATC)_7_ | TCGAGAAGATCTTTAGACTGGTTG | ACACCTGACACTCACATGCT | 213 | low-success-rate |
| S109 | contig35212 | (AAAT)_7_ | AGGAGTAGCGGACAAGTGAT | TGTCACCATGGCAATAAGGGT | 138 | non-amplification |
| S110 | contig35410 | (ATC)_9_ | AAAGACGAGACAGACACGCT | TAGCCCTCGACGAATCAACG | 116 | polymorphism |
| S111 | contig3558 | (AACCT)_8_ | GCATGAACTTCGAACAGGTAGG | ACCAATTGCATAATGGAACCTCA | 219 | polymorphism |
| S112 | contig35775 | (ACTC)_7_ | GATACAAGTCCGTCCGAGGC | CGAGGTCGAAGAGAATACTGCA | 151 | low-success-rate |
| S113 | contig3601 | (AAG)_7_ | GATTGCTGGGCACGGTTTAC | CGGGCATCTTTAACACGGGA | 231 | polymorphism |
| S114 | contig36098 | (ACTCG)_6_ | CAACCTCCCTGGCACAATCA | TTTCGGAGGGAACAGAGCAG | 143 | polymorphism |
| S115 | contig36343 | (ACGTC)_8_ | TGTCTTTCTGAAAGTCTTTCTGGT | TGACGAAACTGTGAACGACAA | 191 | low-success-rate |
| S116 | contig36355 | (ACG)_8_ | CGTTCCGAGATTTGCAGCAA | CAGAATGGGACGGCATCAGA | 124 | polymorphism |
| S117 | contig36374 | (AGG)_7_ | GCTTCCTGTGGAATTTAATCCTCT | CGTGCGAACAATGGCAGAAG | 157 | polymorphism |
| S118 | contig36922 | (AAG)_8_ | CGACATCGACAGTAGGGTGG | AACGTCCGCTCTGTAACACA | 265 | low-success-rate |
| S119 | contig37047 | (ACCCAG)_6_ | GCACATATGCCAGTGACGAA | CACTCTCGAATTATCAGGCCCT | 128 | low-success-rate |
| S123 | contig37713 | (AACCT)_11_ | AGCACTTTCAGGTATACGAGGG | AGCGAGAGTCTACTGCATTGT | 202 | no polymorphism |
| S124 | contig37922 | (AAAT)_9_ | GTACACCATAAGCTGCCATGG | TGACCCTCATTTGCGTATCCT | 207 | low-success-rate |
| S125 | contig37997 | (AAG)_7_ | GATTGTAGGTTGGCCACTATGT | GTCTTTCACCACGGACAGATT | 141 | polymorphism |
| S126 | contig38701 | (AAC)_7_ | AAGCCGAGCATTAACCGGAA | ACAACGCTGCCAGTACCTAC | 145 | low-success-rate |
| S127 | contig38840 | (ACAT)_7_ | GCAGGCGGCTTATTGATCTT | CCTTTAGGACTTCTTGAAGAGCCT | 160 | low-success-rate |
| S128 | contig38886 | (AACCT)_8_ | GAATCGATCAAGACTTTGGCCAG | CATTCCGTCGCAGGGATCAT | 190 | low-success-rate |
| S129 | contig39362 | (AACGAC)_9_ | TGTGTCTTTACCATGTTTCACGT | TGGATTGTTGAAATCAGTAGGGAA | 229 | polymorphism |
| S132 | contig39781 | (AAGT)_7_ | TTGCTCTAACCCGGTGGAAC | GCTGTACTCATTCGTATAGCTCAT | 108 | polymorphism |
| S133 | contig40224 | (AAG)_7_ | GTAACATGCGGAAGTTGGCC | TGTACTCGGACAGGAACGAC | 180 | polymorphism |
| S134 | contig40308 | (AACCT)_9_ | GCGGAGTTGAGTTGCATCAC | CAGCTGATCCAGAGTGGCAA | 285 | polymorphism |
| S135 | contig40341 | (ACT)_14_ | CCGGATGCGGTGATAACTGT | GAACGCAGAGTAGAGACGGG | 173 | polymorphism |
| S136 | contig41258 | (ATCGCC)_20_ | AGTTCACCCGTCGTATTGCA | AATGACGATGACGATGGCGA | 276 | faint bands |
| S137 | contig41523 | (ACCGCC)_13_ | CTCTCGGTAGCTGCGAAGTC | CTGATTCTGCTCCACCTCCC | 182 | low-success-rate |
| S140 | contig42331 | (ACCGTC)_7_ | TGGTCCCGCCAAAGTTAGAG | AATACCACCGTGACCGTCAC | 208 | non-amplification |
| S141 | contig43911 | (AAG)_7_ | GCTGTACGGGCTTCTGACTC | GTGCTAACTGGCTGATTGGC | 98 | polymorphism |
| S142 | contig44747 | (AAG)_7_ | AATGGCTGATTTGTGTCAACGAG | AATCGCGTTACGTAATATGTGGAC | 190 | non-specific amplification |
| S143 | contig45467 | (AAG)_8_ | GGCTGCGCCTTGATTAATCG | TAAATCGTTTCCGCGTTCGC | 136 | low-success-rate |
| S144 | contig45539 | (ATC)_7_ | CATGCGTGCTTCTTAGGACTT | CGACCATTGTTGCCTAGGGA | 174 | polymorphism |
| S145 | contig45811 | (AAG)_7_ | ATCCGACTGTGGAGCACATG | AGTGCGGTTAAGAAGGCTGT | 102 | faint bands |
| S146 | contig46379 | (ACT)_10_ | ATTACGGCGGCTGCTTAGG | GCAACAAGCTATTCTCACGGG | 116 | low-success-rate |
| S147 | contig4644 | (AGC)_7_ | GTTAGCACGTCGCAGAGCTC | GCGCGGAAGTAGTGTTGGTA | 191 | no polymorphism |
| S148 | contig46442 | (ACG)_7_ | ACATGGAATCAGGAGAGCGC | GCAGCTCATCCAACCCTTTC | 108 | polymorphism |
| S149 | contig46948 | (AAG)_8_ | CCATTGGTCTAAGGTAGGGTGG | ATACGTCCATGGTTTCGGCA | 190 | polymorphism |
| S150 | contig47570 | (AACT)_7_ | TGGCACAATGGGCTTATAGC | ACCTGTCACCCACCAAATGT | 196 | polymorphism |
| S151 | contig48635 | (AACCT)_6_ | ACGAGTGTACACCAGTGTAACC | TCGAACGACGACCTGCATTT | 99 | low-success-rate |
| S152 | contig4878 | (ACTGAG)_7_ | GCCAATGCTTGTAGGCAGGA | CGACAGTCTCCCATCCCTCT | 194 | low-success-rate |
| S155 | contig49892 | (AACCT)_7_ | GGGTCTAAATTTCCAGGAATAGGG | CCAGCCGGATAAGATGGTCA | 133 | faint bands |
| S156 | contig50317 | (AAG)_7_ | CCGGTAATTCTCATTAATTCCGGT | TAACTGGCTGATTGGCGCAA | 112 | non-specific amplification |
| S157 | contig51318 | (AAG)_7_ | CAGAGGATCGCGCAAGAAGT | CTTGATTTCACTTCGCGCCC | 155 | non-amplification |
| S158 | contig51836 | (AAG)_8_ | CCTGAGCACCCAACTCGAAG | ACACTTTCCCGTCACGTCAC | 250 | low-success-rate |
| S159 | contig51963 | (AATG)_7_ | GTATAGAGGTGGAGGCGTGC | TGACAAACACAGTGCGGTCT | 198 | polymorphism |
| S160 | contig52200 | (AAAC)_9_ | AGATGCGTTCAGACTCTGCC | GTTGTCCTGACGTTAGCGGA | 153 | polymorphism |
| S161 | contig54435 | (AAAT)_8_ | CAATCATCGGGACCTCCTCG | GTTGTTGCTCGCATGCTGAG | 157 | low-success-rate |
| S162 | contig5473 | (AAG)_8_ | CAGGGAGGGTTTCTAACAGCA | GCCTTGGTTCTAAGTACAGTAGAC | 166 | polymorphism |
| S163 | contig55749 | (AACCT)_6_ | ATTGGCAGACTCAGCTGTGT | GGGTCGACTATTTAGGGACATTAC | 292 | non-specific amplification |
| S164 | contig55925 | (ACGTC)_6_ | ACGCCTACACGGGAAATGTT | GAGGAACACCCGAATGTGGT | 345 | polymorphism |
| S165 | contig5675 | (AAC)_8_ | AACGACGATGACCAACAAAGA | CGGAACAGTTGTGACCTGGG | 112 | faint bands |
| S166 | contig56860 | (ATC)_7_ | TAGAAGTCACCAGCGAGCAG | TCAATTCCTCCACGTGCCTT | 114 | non-specific amplification |
| S167 | contig57302 | (AAG)_10_ | TCATGCGGAATTGTTTCGAACT | AAGTACTAACTGGCAGATTGGC | 142 | low-success-rate |
| S168 | contig58132 | (AATGAC)_6_ | TGGATATGACAGCCTGCAACA | CGTTGCAATTACTCGTACCAAAC | 290 | low-success-rate |
| S171 | contig5865 | (ACGAGC)_13_ | ACTTGCAAGCACTCGCAAAG | TCGAGTGGAGGCATTTGCAA | 254 | non-amplification |
| S174 | contig59194 | (AACGAT)_7_ | CGTCGTCATCGCAATCATCG | CGATGTCGTCAAAGATCACGA | 208 | low-success-rate |
| S175 | contig61288 | (AAG)_7_ | AACATTATCATTGAGTCCGCCA | AAAGTATCGAGCCCAAACGG | 209 | non-specific amplification |
| S176 | contig62635 | (ATC)_8_ | GTGCCGAACGCGTATTTCTC | CCGGGCCATTCTATCAATCCA | 152 | polymorphism |
| S177 | contig63082 | (AAG)_7_ | GCTGCCTTCAAGGATTCTCG | ACTGGTCGTATAGCCAAGGC | 143 | faint bands |
| S178 | contig64806 | (AAG)_7_ | GGAAGATGGGAGAGTGGTGG | CCGCAATCCCATTCTCACCA | 191 | non-amplification |
| S179 | contig65017 | (AGG)_8_ | CCAGCAGTTGGTAGATACAAGTG | CTGTGGAAGTTGACTCCGTTT | 141 | low-success-rate |
| S181 | contig6503 | (AAGTGC)_6_ | TGCACTTCTTTCCCTGCACA | ACTCGTAGCAAGATGTCGGTC | 286 | no polymorphism |
| S185 | contig65600 | (AAG)_7_ | CTCGTAGGTTCAGCGGCC | CGAACACCTTTGCAAGACGA | 158 | polymorphism |
| S186 | contig65867 | (AAG)_14_ | CACATGCTTCCGCCAATCAG | TCAATTGGCTCTGGTCGCAT | 128 | non-amplification |
| S187 | contig6667 | (ACT)_13_ | GCAAATGCCTACCTTGCCTA | GCAACAACATCACAAGTATCCTCA | 248 | faint bands |
| S188 | contig7029 | (ACAG)_10_ | CTGACCACCGAACACTGTCA | TTCTACAGAGGAGGTGCGGA | 127 | polymorphism |
| S190 | contig704 | (ACTG)_7_ | CCGCGATCGTTCAGTAGTGT | CGCACTTGTGGAAGAGAAGC | 193 | polymorphism |
| S191 | contig709 | (AAATT)_7_ | GAGCTTCACCATCAGCTTCTT | TCATCAGCAACTGAGAGCCC | 202 | non-amplification |
| S193 | contig71030 | (AACCT)_7_ | TCGACGTGCTTGAAGAACCA | GCCCACCAAACCTTCCACTT | 187 | low-success-rate |
| S194 | contig7159 | (AGAT)_12_ | CCGGGACAGATCTTGACACT | CGTTGCTACAGAAATTCACCAGT | 138 | low-success-rate |
| S195 | contig72451 | (AATC)_8_ | AGGAGCGCTTAGTATTCTCAGT | GTGACTGAGAGTGACTGAGACA | 241 | faint bands |
| S196 | contig73304 | (AAG)_7_ | TTCAAGTGGCTCCAGACGAC | CGTTGAGGCGCTCACATTTC | 174 | no polymorphism |
| S197 | contig73309 | (AAAT)_10_ | AGCCAACGATCTTCTTCGGG | CTCACTCTGACGATTGCGAA | 188 | low-success-rate |
| S198 | contig7344 | (AAC)_7_ | GAGGCAACCTCTCGTTTCGA | CTGTTGCAGAGACCCACACT | 154 | no polymorphism |
| S199 | contig75406 | (AATGAC)_6_ | GGTCGTCACTGTGATTCGGA | CTCGGCTTCATTCGTTGTGC | 185 | low-success-rate |
| S200 | contig76240 | (ACGTC)_8_ | CATTTCGCCTGGAACTCGTTC | ATAACTTTCGTAACGCGCCG | 341 | low-success-rate |
| S201 | contig77227 | (AATG)_9_ | TGTCATCGCATTGCTGATGG | TCGTGTTGGGTGTAACAGGG | 159 | low-success-rate |
| S202 | contig8240 | (AAG)_7_ | TCACGTGACCAGTCTAACGC | ACCGATGAATTCAGACGTCCC | 166 | polymorphism |
| S203 | contig8363 | (AATT)_9_ | AGCTAGTACTTACGCATTTGTTGA | ACGACCGAATGAATCTCTAAGTGT | 199 | faint bands |
| S204 | contig84280 | (ACAGCG)_7_ | TACACAGCAACAGCAACAGC | CTGTCCCTGTGTCGCTGTC | 161 | non-amplification |
| S205 | contig8604 | (ATC)_13_ | TCAGCAAAGTTCCTAGGCAGG | CCCATCCATCATGCGCAGA | 142 | polymorphism |
| S206 | contig8638 | (ACC)_8_ | AAATGCTGATAGGGCGAGGC | GAGGCGGAGGTGGAGGTG | 176 | polymorphism |
| S207 | contig8704 | (ACC)_7_ | TCAGCAGGCGAGTAAGTTTGT | CAGCTGAGTATGGACCACCG | 139 | polymorphism |
| S208 | contig8722 | (AACCT)_8_ | CAAATCCGATCGTCCAACGC | GATCGCAATGCATGGAGCTG | 192 | low-success-rate |
| S209 | contig8869 | (AAC)_11_ | CTATGCCAAACAGCGAGTGC | CTCCTTAGGCGCATCGAAGT | 247 | non-amplification |
| S210 | contig8880 | (AAG)_10_ | TCAACTCTACACGGTGGGAAC | ACCAGTTAGAACCTTGCTTGAGA | 240 | low-success-rate |
| S211 | contig8992 | (ACG)_7_ | TTCCAGGCTGTCACGAAGAG | ACGTGCAGTGTAGACATGGT | 246 | faint bands |
| S212 | contig9050 | (AAG)_9_ | TACACGCGTCAGTATCGGAC | CCCTCTTCGAGCACTCCAAT | 156 | faint bands |
| S213 | contig9056 | (AATG)_11_ | GCATCGCCCAATGTTTAACGT | GTCTCCACTTAGACGAGCGTC | 190 | polymorphism |
| S214 | contig9438 | (AAG)_7_ | TGCATTTGTGGTGCATCGAA | GAACTTGTTAATCGCGGTCTGA | 295 | polymorphism |

- **Table S3.** Information of 69 microsatellite markers developed for *Acantholyda posticalis*.

| Locus | Motif | Forward primer 5’=>3’ | Reverse primer 5’=>3’ | Fluorescent label | PCR product size (bp) |
| --- | --- | --- | --- | --- | --- |
| S1 | (ACGG)_7_ | CTCAGGGAACATTGCTGTGC | TTGCTCCTGTCCGTTAGCC | FAM | 98-110 |
| S2 | (ATC)_7_ | CGCTCGTCTCATAGCCTGAA | TGCAACATTGTGAAGGGAGTAC | FAM | 173-179 |
| S4 | (ACT)_7_ | GTGCTTTCTTTGTCAGGCCG | TGAACCATGTACCTCCAAGCT | FAM | 135-138 |
| S5 | (AACCT)_8_ | ATCGACCGTTTGACAAATATGAGT | CGCAAGCCTTAGGATGTGGA | HEX | 202-222 |
| S10 | (ACT)_9_ | ACCTCAGTGCTTCTACCTGG | TCGCGCAGTTTCACGGATAG | HEX | 193-196 |
| S11 | (ACC)_8_ | CCAGGCTCTGCGGATGTT | GGTGATGTACCGAAGTTTGAGA | FAM | 159-168 |
| S16 | (AAC)_8_ | CCTCTGCGAGATCAGCAACA | TCACCTGACAACGGCTTTCT | FAM | 127-130 |
| S18 | (ATC)_10_ | CGTCCCTCCTATAACCGTACA | ACCTCGGTTTCACAGCTTCA | FAM | 156-168 |
| S20 | (AAG)_7_ | GCATCAGACTGCACAAACGC | ACGGAAGATCGAAGCAGACG | HEX | 222-225 |
| S21 | (AAG)_7_ | AATCAACTGGCTGATTGGCG | TCCCTAGATTCGGAGCGATG | FAM | 146-158 |
| S24 | (AAG)_7_ | GGCTCAAGTCACGTCGTGTA | GCACTTCAACTGCATGATTGGT | FAM | 170-176 |
| S25 | (AAG)_8_ | AGGCTGATCGTAAACCTCGT | GAGTGGGCGCGAGATAAAGT | FAM | 157-160 |
| S27 | (AAG)_9_ | AGCGAATAGTCAACTCCAACCT | AGACGTCGAAATCGGTGTATTG | FAM | 116-125 |
| S28 | (AAG)_8_ | TGGCAATGAGTTACGAGATCG | CGAACAAATCTGTGCACCTCC | HEX | 220-241 |
| S29 | (AAG)_9_ | TATGTGCCAAATGCCGAGGT | CACCCACATTTCGGAGAGCT | HEX | 306-327 |
| S30 | (AAG)_8_ | CGCCTCGAATAACGTCAATCC | GCGCGTGGAATGTAAATCCG | HEX | 2761-282 |
| S36* | (AAG)_7_ | AGATCTGTGAGCCGTTCGC | CTCGAGAGTGCGCCTATGTT | FAM | 156-162 |
| S39 | (AAG)_7_ | AATAATGGTGTCGGCCAGCT | CCCTCCCAAGCTACACTCTTC | HEX | 222-240 |
| S41* | (AAG)_9_ | TCGTTTCCTTGACAGGTGTCT | GGATGCCTGGTCACGTTACT | HEX | 242-263 |
| S42 | (AAG)_7_ | TGGTTGACACTGGTTTCTTTGC | GTCAAACACGATTCTCGAGACA | FAM | 156-168 |
| S47 | (AATG)_7_ | GCCAAGTTATCTGCGCCATC | ATCGACTTCTTCAGACAATCTGTT | FAM | 114-162 |
| S48* | (AGC)_8_ | CTACACAGCAGACAGGAGGG | GTTTGGTCAGAACAGACGGG | HEX | 242-257 |
| S50 | (AAAT)_7_ | TACTCGCACTTGACCGGTTT | CCGCCATTCCACCACCTATT | HEX | 256-272 |
| S55 | (AATGAC)_8_ | TGACTGACTTTATGTGGAATCCCT | ACGTCAACAGCGGCTCATTA | HEX | 309-387 |
| S62* | (AACCT)_9_ | TCCTTCAAGGACTTAGCGTTGA | CCTACCGGTTCACACCATCC | HEX | 216-251 |
| S63* | (ACT)_9_ | AAACACCCTGTACAAGGCGC | GGCAACAACCAACTCAAGGG | FAM | 157-163 |
| S64 | (AAAT)_7_ | ATTCCATAATAACGCTTGCCGA | GCTTAGCACCATTTACGGCC | FAM | 145-169 |
| S74* | (AAG)_13_ | GTTGGCATTAGCAAGCTGGG | CTACAATCCGGCTGGCTGTT | FAM | 164-221 |
| S76 | (AAC)_7_ | AATACGGAACTCAGCGAGGC | TGCACCCGCTATCGAGTAAC | HEX | 238-241 |
| S85* | (ATC)_9_ | AACTGTGTTGCGCCTACTGA | GCCAATTAGCTGCAGATGGC | FAM | 119-155 |
| S90 | (AAG)_7_ | TCGAATACTCGCTCCAAGCC | CATCAAGCGTCAAACCGAGC | HEX | 208-211 |
| S92 | (AACCT)_8_ | CGCGGATGATTAATAGCGCG | TGTTCCGGAGAGTCAAGTCC | FAM | 170-205 |
| S94 | (AATCG)_6_ | CACAACTCGCGTCATTCGAC | TCGAGATTCGATTCGGCCC | HEX | 196-206 |
| S98 | (AGC)_7_ | TCTTCGTGTTCCTGGTCTGG | AACAGGCTTCGGAGTACAGC | FAM | 156-159 |
| S99 | (ATC)_7_ | ACGTAGGATTCCACAGTGCG | CTATGGACTTGCCTGCACCT | HEX | 230-243 |
| S100 | (ACCT)_8_ | ACCTGGCAAGAGTCTGAAAGT | ACAGGCGTCACTTGATCACT | HEX | 298-302 |
| S103 | (AAC)_7_ | GGTGTCAGCACGACTCAGAA | CCGGTCCTCGCTTTGTAGTT | HEX | 210-216 |
| S107 | (AAG)_10_ | TGCACTATCTTCAATCTCGGACA | GAAGGAGGAACAGTGGCGTT | HEX | 233-239 |
| S110 | (ATC)_9_ | AAAGACGAGACAGACACGCT | TAGCCCTCGACGAATCAACG | FAM | 124-133 |
| S111* | (AACCT)_8_ | GCATGAACTTCGAACAGGTAGG | ACCAATTGCATAATGGAACCTCA | HEX | 220-245 |
| S113* | (AAG)_7_ | GATTGCTGGGCACGGTTTAC | CGGGCATCTTTAACACGGGA | HEX | 246-249 |
| S114 | (ACTCG)_6_ | CAACCTCCCTGGCACAATCA | TTTCGGAGGGAACAGAGCAG | FAM | 149-179 |
| S116 | (ACG)_8_ | CGTTCCGAGATTTGCAGCAA | CAGAATGGGACGGCATCAGA | FAM | 140-143 |
| S117 | (AGG)_7_ | GCTTCCTGTGGAATTTAATCCTCT | CGTGCGAACAATGGCAGAAG | FAM | 168-177 |
| S125 | (AAG)_7_ | GATTGTAGGTTGGCCACTATGT | GTCTTTCACCACGGACAGATT | FAM | 155-161 |
| S129 | (AACGAC)_9_ | TGTGTCTTTACCATGTTTCACGT | TGGATTGTTGAAATCAGTAGGGAA | HEX | 220-292 |
| S132* | (AAGT)_7_ | TTGCTCTAACCCGGTGGAAC | GCTGTACTCATTCGTATAGCTCAT | FAM | 115-127 |
| S133 | (AAG)_7_ | GTAACATGCGGAAGTTGGCC | TGTACTCGGACAGGAACGAC | HEX | 191-203 |
| S134 | (AACCT)_9_ | GCGGAGTTGAGTTGCATCAC | CAGCTGATCCAGAGTGGCAA | HEX | 298-308 |
| S135* | (ACT)_14_ | CCGGATGCGGTGATAACTGT | GAACGCAGAGTAGAGACGGG | HEX | 172-223 |
| S141 | (AAG)_7_ | GCTGTACGGGCTTCTGACTC | GTGCTAACTGGCTGATTGGC | FAM | 111-114 |
| S144* | (ATC)_7_ | CATGCGTGCTTCTTAGGACTT | CGACCATTGTTGCCTAGGGA | HEX | 191-194 |
| S148 | (ACG)_7_ | ACATGGAATCAGGAGAGCGC | GCAGCTCATCCAACCCTTTC | FAM | 108-123 |
| S149 | (AAG)_8_ | CCATTGGTCTAAGGTAGGGTGG | ATACGTCCATGGTTTCGGCA | HEX | 202-256 |
| S150 | (AACT)_7_ | TGGCACAATGGGCTTATAGC | ACCTGTCACCCACCAAATGT | HEX | 205-217 |
| S159 | (AATG)_7_ | GTATAGAGGTGGAGGCGTGC | TGACAAACACAGTGCGGTCT | HEX | 216-224 |
| S160 | (AAAC)_9_ | AGATGCGTTCAGACTCTGCC | GTTGTCCTGACGTTAGCGGA | FAM | 144-176 |
| S162 | (AAG)_8_ | CAGGGAGGGTTTCTAACAGCA | GCCTTGGTTCTAAGTACAGTAGAC | FAM | 169-211 |
| S164 | (ACGTC)_6_ | ACGCCTACACGGGAAATGTT | GAGGAACACCCGAATGTGGT | HEX | 353-388 |
| S176 | (ATC)_8_ | GTGCCGAACGCGTATTTCTC | CCGGGCCATTCTATCAATCCA | FAM | 166-172 |
| S185 | (AAG)_7_ | CTCGTAGGTTCAGCGGCC | CGAACACCTTTGCAAGACGA | FAM | 172-178 |
| S188 | (ACAG)_10_ | CTGACCACCGAACACTGTCA | TTCTACAGAGGAGGTGCGGA | FAM | 132-156 |
| S190 | (ACTG)_7_ | CCGCGATCGTTCAGTAGTGT | CGCACTTGTGGAAGAGAAGC | HEX | 206-222 |
| S202 | (AAG)_7_ | TCACGTGACCAGTCTAACGC | ACCGATGAATTCAGACGTCCC | FAM | 182-194 |
| S205 | (ATC)_13_ | TCAGCAAAGTTCCTAGGCAGG | CCCATCCATCATGCGCAGA | FAM | 145-166 |
| S206 | (ACC)_8_ | AAATGCTGATAGGGCGAGGC | GAGGCGGAGGTGGAGGTG | HEX | 188-191 |
| S207 | (ACC)_7_ | TCAGCAGGCGAGTAAGTTTGT | CAGCTGAGTATGGACCACCG | FAM | 152-158 |
| S213 | (AATG)_11_ | GCATCGCCCAATGTTTAACGT | GTCTCCACTTAGACGAGCGTC | HEX | 200-216 |
| S214* | (AAG)_7_ | TGCATTTGTGGTGCATCGAA | GAACTTGTTAATCGCGGTCTGA | HEX | 308-314 |

Note: The “*” denotes loci that were removed for violating HWE and showing high null allele frequencies.

- **Table S5.** Frequency distribution of different microsatellite types in *Acantholyda posticalis*.

| Motif | Total counts | Average length (bp) | Relative abundance (loci/Mb) | Relative density (Kb/Mb) | Motif length(bp) | | | | | | | | |
| --- | --- | --- | --- | --- | --- | --- | --- | --- | --- | --- | --- | --- | --- |
|  |  |  |  |  | 12-35 | 36-45 | 46-55 | 56-65 | 66-75 | 76-85 | 86-95 | 96-105 | >105 |
| Mono | 55429 | 14.75 | 80.11 | 0.12 | 55031 | 133 | 73 | 151 | 18 | 11 | 4 | 5 | 3 |
| Di | 20609 | 24.06 | 29.78 | 0.72 | 16801 | 2259 | 1010 | 286 | 101 | 59 | 27 | 22 | 44 |
| Tri | 11590 | 26.13 | 16.75 | 0.44 | 9744 | 1067 | 372 | 187 | 111 | 32 | 18 | 16 | 43 |
| Tetra | 3801 | 33.63 | 5.49 | 0.18 | 2846 | 492 | 135 | 122 | 47 | 43 | 22 | 29 | 65 |
| Penta | 713 | 39.11 | 1.03 | 0.04 | 520 | 70 | 38 | 19 | 9 | 13 | 4 | 1 | 39 |
| Hexa | 1734 | 67.01 | 2.51 | 0.17 | 388 | 416 | 190 | 61 | 118 | 98 | 31 | 73 | 359 |
| Total | 93876 | 20.12 | 135.67 | 2.73 | 85330 | 4437 | 1818 | 826 | 404 | 256 | 106 | 146 | 553 |

Note: Mono, mononucleotide repeats; Di, dinucleotide repeats; Tri, trinucleotide repeats; Tetra, tetranucleotide repeats; Penta, pentanucleotide repeats; Hexa, hexanucleotide repeats.

- **Table S6**. Microsatellite motif types in *Acantholyda posticalis*.

| Motif type | Motif length | Total counts | Total length (bp) | Average length (bp) | Relative abundance (loci/Mb) | Relative density (bp/Mb) | Frequency (%) |
| --- | --- | --- | --- | --- | --- | --- | --- |
| C | 1 | 29975 | 448996 | 14.98 | 43.320 | 648.89 | 31.930 |
| A | 1 | 25454 | 368631 | 14.48 | 36.786 | 532.74 | 27.114 |
| AT | 2 | 11857 | 306322 | 25.83 | 17.136 | 442.70 | 12.630 |
| AAT | 3 | 9401 | 252258 | 26.83 | 13.586 | 364.56 | 10.014 |
| AG | 2 | 5290 | 113908 | 21.53 | 7.645 | 164.62 | 5.635 |
| AC | 2 | 3325 | 73410 | 22.08 | 4.805 | 106.09 | 3.542 |
| AAG | 3 | 1372 | 30234 | 22.04 | 1.983 | 43.69 | 1.462 |
| AAAT | 4 | 1044 | 31396 | 30.07 | 1.509 | 45.37 | 1.112 |
| ACAG | 4 | 865 | 24504 | 28.33 | 1.250 | 35.41 | 0.921 |
| AATGAC | 6 | 391 | 26820 | 68.59 | 0.565 | 38.76 | 0.417 |
| AATG | 4 | 375 | 14504 | 38.68 | 0.542 | 20.96 | 0.399 |
| ATAC | 4 | 263 | 8668 | 32.96 | 0.380 | 12.53 | 0.280 |
| ACGC | 4 | 261 | 15964 | 61.16 | 0.377 | 23.07 | 0.278 |
| AACCT | 5 | 236 | 8165 | 34.60 | 0.341 | 11.80 | 0.251 |
| AATGAT | 6 | 229 | 18480 | 80.70 | 0.331 | 26.71 | 0.244 |
| ATC | 3 | 225 | 6459 | 28.71 | 0.325 | 9.33 | 0.240 |
| ACTC | 4 | 212 | 8048 | 37.96 | 0.306 | 11.63 | 0.226 |
| ATAG | 4 | 203 | 6840 | 33.69 | 0.294 | 9.89 | 0.216 |
| ACT | 3 | 165 | 4218 | 25.56 | 0.239 | 6.10 | 0.176 |
| AAC | 3 | 160 | 3711 | 23.19 | 0.231 | 5.36 | 0.170 |
| CG | 2 | 137 | 2202 | 16.07 | 0.198 | 3.18 | 0.146 |
| ACACGC | 6 | 122 | 8958 | 73.43 | 0.176 | 12.95 | 0.130 |
| AAAC | 4 | 113 | 3068 | 27.15 | 0.163 | 4.43 | 0.120 |
| ACGTC | 5 | 89 | 5370 | 60.34 | 0.129 | 7.76 | 0.095 |
| AGC | 3 | 81 | 1752 | 21.63 | 0.117 | 2.53 | 0.086 |
| AATC | 4 | 80 | 2552 | 31.90 | 0.116 | 3.69 | 0.085 |
| ACAGTG | 6 | 75 | 4866 | 64.88 | 0.108 | 7.03 | 0.080 |
| AACG | 4 | 74 | 2820 | 38.11 | 0.107 | 4.08 | 0.079 |
| AATGGC | 6 | 71 | 5232 | 73.69 | 0.103 | 7.56 | 0.076 |
| AATTC | 5 | 67 | 2330 | 34.78 | 0.097 | 3.37 | 0.071 |
| ACG | 3 | 66 | 1701 | 25.77 | 0.095 | 2.46 | 0.070 |
| AATT | 4 | 65 | 1688 | 25.97 | 0.094 | 2.44 | 0.069 |
| ATCGTC | 6 | 64 | 5322 | 83.16 | 0.092 | 7.69 | 0.068 |
| ACGG | 4 | 60 | 2020 | 33.67 | 0.087 | 2.92 | 0.064 |
| AACGAC | 6 | 58 | 4014 | 69.21 | 0.084 | 5.80 | 0.062 |
| ACTCTC | 6 | 57 | 3042 | 53.37 | 0.082 | 4.40 | 0.061 |
| AGG | 3 | 51 | 1068 | 20.94 | 0.074 | 1.54 | 0.054 |
| ACC | 3 | 46 | 1035 | 22.50 | 0.067 | 1.50 | 0.049 |
| ACTG | 4 | 42 | 1228 | 29.24 | 0.061 | 1.77 | 0.045 |
| ACAGCG | 6 | 42 | 3078 | 73.29 | 0.061 | 4.45 | 0.045 |
| AATAG | 5 | 38 | 1335 | 35.13 | 0.055 | 1.93 | 0.040 |
| AACCGT | 6 | 35 | 1680 | 48.00 | 0.051 | 2.43 | 0.037 |
| AATAT | 5 | 32 | 1010 | 31.56 | 0.046 | 1.46 | 0.034 |
| AAAG | 4 | 28 | 736 | 26.29 | 0.040 | 1.06 | 0.030 |
| ATCC | 4 | 24 | 1004 | 41.83 | 0.035 | 1.45 | 0.026 |
| CCG | 3 | 23 | 453 | 19.70 | 0.033 | 0.65 | 0.025 |
| AAGG | 4 | 23 | 828 | 36.00 | 0.033 | 1.20 | 0.025 |
| ACAGAG | 6 | 21 | 1542 | 73.43 | 0.030 | 2.23 | 0.022 |
| AATAC | 5 | 20 | 655 | 32.75 | 0.029 | 0.95 | 0.021 |
| ACCGTG | 6 | 20 | 1152 | 57.60 | 0.029 | 1.66 | 0.021 |
| AAAAT | 5 | 19 | 560 | 29.47 | 0.027 | 0.81 | 0.020 |
| AACCTC | 6 | 19 | 774 | 40.74 | 0.027 | 1.12 | 0.020 |
| AGAGGG | 6 | 19 | 840 | 44.21 | 0.027 | 1.21 | 0.020 |
| ACCGCC | 6 | 18 | 792 | 44.00 | 0.026 | 1.14 | 0.019 |
| ACGAG | 5 | 17 | 430 | 25.29 | 0.025 | 0.62 | 0.018 |
| AAACTC | 6 | 17 | 1104 | 64.94 | 0.025 | 1.60 | 0.018 |
| AATGTC | 6 | 17 | 984 | 57.88 | 0.025 | 1.42 | 0.018 |
| AATGC | 5 | 15 | 405 | 27.00 | 0.022 | 0.59 | 0.016 |
| AATATC | 6 | 15 | 966 | 64.40 | 0.022 | 1.40 | 0.016 |
| ACAGTC | 6 | 15 | 1044 | 69.60 | 0.022 | 1.51 | 0.016 |
| AGCC | 4 | 14 | 464 | 33.14 | 0.020 | 0.67 | 0.015 |
| AATCG | 5 | 14 | 615 | 43.93 | 0.020 | 0.89 | 0.015 |
| ATATAC | 6 | 14 | 534 | 38.14 | 0.020 | 0.77 | 0.015 |
| ACCCG | 5 | 13 | 600 | 46.15 | 0.019 | 0.87 | 0.014 |
| AATGAG | 6 | 13 | 1176 | 90.46 | 0.019 | 1.70 | 0.014 |
| ACCGTC | 6 | 13 | 810 | 62.31 | 0.019 | 1.17 | 0.014 |
| ATGACC | 6 | 13 | 636 | 48.92 | 0.019 | 0.92 | 0.014 |
| AATCTC | 6 | 12 | 1290 | 107.50 | 0.017 | 1.86 | 0.013 |
| AAATT | 5 | 11 | 320 | 29.09 | 0.016 | 0.46 | 0.012 |
| ATCAGC | 6 | 11 | 678 | 61.64 | 0.016 | 0.98 | 0.012 |
| AAGT | 4 | 10 | 296 | 29.60 | 0.015 | 0.43 | 0.011 |
| AATCT | 5 | 10 | 265 | 26.50 | 0.014 | 0.38 | 0.011 |
| AACAGT | 6 | 10 | 666 | 66.60 | 0.014 | 0.96 | 0.011 |
| AACGAT | 6 | 10 | 642 | 64.20 | 0.014 | 0.93 | 0.011 |
| AACGGT | 6 | 10 | 420 | 42.00 | 0.015 | 0.61 | 0.011 |
| AAGGAC | 6 | 10 | 786 | 78.60 | 0.015 | 1.14 | 0.011 |
| ATATAG | 6 | 10 | 474 | 47.40 | 0.015 | 0.69 | 0.011 |
| ACCC | 4 | 9 | 228 | 25.33 | 0.013 | 0.33 | 0.010 |
| AAATG | 5 | 8 | 380 | 47.50 | 0.012 | 0.55 | 0.009 |
| AAAGAG | 6 | 8 | 804 | 100.50 | 0.012 | 1.16 | 0.009 |
| ACGCGC | 6 | 8 | 372 | 46.50 | 0.012 | 0.54 | 0.009 |
| ACTGCG | 6 | 8 | 468 | 58.50 | 0.012 | 0.68 | 0.009 |
| ATAGTC | 6 | 8 | 288 | 36.00 | 0.012 | 0.42 | 0.009 |
| AACGC | 5 | 7 | 715 | 102.14 | 0.010 | 1.03 | 0.007 |
| ACACG | 5 | 7 | 765 | 109.29 | 0.010 | 1.11 | 0.007 |
| ACGCG | 5 | 7 | 190 | 27.14 | 0.010 | 0.27 | 0.007 |
| AAATAT | 6 | 7 | 396 | 56.57 | 0.010 | 0.57 | 0.007 |
| ACTGCC | 6 | 7 | 396 | 56.57 | 0.010 | 0.57 | 0.007 |
| AACT | 4 | 6 | 148 | 24.67 | 0.009 | 0.21 | 0.006 |
| ACCG | 4 | 6 | 180 | 30.00 | 0.009 | 0.26 | 0.006 |
| ACCT | 4 | 6 | 160 | 26.67 | 0.009 | 0.23 | 0.006 |
| ACTCC | 5 | 6 | 495 | 82.50 | 0.009 | 0.72 | 0.006 |
| AAGCAC | 6 | 6 | 456 | 76.00 | 0.009 | 0.66 | 0.006 |
| AATAAC | 6 | 6 | 486 | 81.00 | 0.009 | 0.70 | 0.006 |
| ACCCAG | 6 | 6 | 216 | 36.00 | 0.009 | 0.31 | 0.006 |
| ACGAGC | 6 | 6 | 396 | 66.00 | 0.009 | 0.57 | 0.006 |
| AGAGCG | 6 | 6 | 324 | 54.00 | 0.009 | 0.47 | 0.006 |
| ATACCG | 6 | 6 | 258 | 43.00 | 0.009 | 0.37 | 0.006 |
| ATACGC | 6 | 6 | 336 | 56.00 | 0.009 | 0.49 | 0.006 |
| AAATC | 5 | 5 | 290 | 58.00 | 0.007 | 0.42 | 0.005 |
| AATCC | 5 | 5 | 135 | 27.00 | 0.007 | 0.20 | 0.005 |
| AATGT | 5 | 5 | 155 | 31.00 | 0.007 | 0.22 | 0.005 |
| ACTCG | 5 | 5 | 135 | 27.00 | 0.007 | 0.20 | 0.005 |
| AAACAC | 6 | 5 | 150 | 30.00 | 0.007 | 0.22 | 0.005 |
| AACGAG | 6 | 5 | 270 | 54.00 | 0.007 | 0.39 | 0.005 |
| AACGGC | 6 | 5 | 300 | 60.00 | 0.007 | 0.43 | 0.005 |
| AACGTC | 6 | 5 | 228 | 45.60 | 0.007 | 0.33 | 0.005 |
| AATCAT | 6 | 5 | 570 | 114.00 | 0.007 | 0.82 | 0.005 |
| AATGGT | 6 | 5 | 156 | 31.20 | 0.007 | 0.23 | 0.005 |
| ACACAG | 6 | 5 | 348 | 69.60 | 0.007 | 0.50 | 0.005 |
| ATACAC | 6 | 5 | 180 | 36.00 | 0.007 | 0.26 | 0.005 |
| ATCGCC | 6 | 5 | 318 | 63.60 | 0.007 | 0.46 | 0.005 |
| AGGC | 4 | 4 | 96 | 24.00 | 0.006 | 0.14 | 0.004 |
| AAACC | 5 | 4 | 140 | 35.00 | 0.006 | 0.20 | 0.004 |
| AATGG | 5 | 4 | 135 | 33.75 | 0.006 | 0.20 | 0.004 |
| ACCCC | 5 | 4 | 180 | 45.00 | 0.006 | 0.26 | 0.004 |
| ACGGG | 5 | 4 | 170 | 42.50 | 0.006 | 0.25 | 0.004 |
| AGCCG | 5 | 4 | 175 | 43.75 | 0.006 | 0.25 | 0.004 |
| AAAGAC | 6 | 4 | 420 | 105.00 | 0.006 | 0.61 | 0.004 |
| AATATT | 6 | 4 | 126 | 31.50 | 0.006 | 0.18 | 0.004 |
| AATGCC | 6 | 4 | 162 | 40.50 | 0.006 | 0.23 | 0.004 |
| ACTCTG | 6 | 4 | 144 | 36.00 | 0.006 | 0.21 | 0.004 |
| ACTGAG | 6 | 4 | 138 | 34.50 | 0.006 | 0.20 | 0.004 |
| ATGTCC | 6 | 4 | 348 | 87.00 | 0.006 | 0.50 | 0.004 |
| AACC | 4 | 3 | 72 | 24.00 | 0.004 | 0.10 | 0.003 |
| AAGC | 4 | 3 | 80 | 26.67 | 0.005 | 0.12 | 0.003 |
| AGGG | 4 | 3 | 96 | 32.00 | 0.004 | 0.14 | 0.003 |
| ATCG | 4 | 3 | 84 | 28.00 | 0.004 | 0.12 | 0.003 |
| AAAAG | 5 | 3 | 75 | 25.00 | 0.004 | 0.11 | 0.003 |
| AAACG | 5 | 3 | 130 | 43.33 | 0.004 | 0.19 | 0.003 |
| AAGCC | 5 | 3 | 75 | 25.00 | 0.004 | 0.11 | 0.003 |
| ATACC | 5 | 3 | 110 | 36.67 | 0.004 | 0.16 | 0.003 |
| AAAAAT | 6 | 3 | 102 | 34.00 | 0.004 | 0.15 | 0.003 |
| AAACCG | 6 | 3 | 114 | 38.00 | 0.004 | 0.16 | 0.003 |
| AACCCC | 6 | 3 | 126 | 42.00 | 0.004 | 0.18 | 0.003 |
| AATGTT | 6 | 3 | 102 | 34.00 | 0.004 | 0.15 | 0.003 |
| AATTAT | 6 | 3 | 102 | 34.00 | 0.004 | 0.15 | 0.003 |
| ACAGGT | 6 | 3 | 90 | 30.00 | 0.004 | 0.13 | 0.003 |
| ACCACG | 6 | 3 | 336 | 112.00 | 0.004 | 0.49 | 0.003 |
| ACCCTC | 6 | 3 | 240 | 80.00 | 0.004 | 0.35 | 0.003 |
| ACCTCC | 6 | 3 | 114 | 38.00 | 0.004 | 0.16 | 0.003 |
| ACTCCC | 6 | 3 | 156 | 52.00 | 0.004 | 0.23 | 0.003 |
| AGAGGC | 6 | 3 | 168 | 56.00 | 0.004 | 0.24 | 0.003 |
| ATACTG | 6 | 3 | 90 | 30.00 | 0.004 | 0.13 | 0.003 |
| AGCG | 4 | 2 | 52 | 26.00 | 0.003 | 0.08 | 0.002 |
| AAAAC | 5 | 2 | 50 | 25.00 | 0.003 | 0.07 | 0.002 |
| AAACT | 5 | 2 | 55 | 27.50 | 0.003 | 0.08 | 0.002 |
| AACGT | 5 | 2 | 70 | 35.00 | 0.003 | 0.10 | 0.002 |
| AACTC | 5 | 2 | 60 | 30.00 | 0.003 | 0.09 | 0.002 |
| AACTG | 5 | 2 | 65 | 32.50 | 0.003 | 0.09 | 0.002 |
| AAGTG | 5 | 2 | 60 | 30.00 | 0.003 | 0.09 | 0.002 |
| ACCCT | 5 | 2 | 85 | 42.50 | 0.003 | 0.12 | 0.002 |
| ACCGT | 5 | 2 | 50 | 25.00 | 0.003 | 0.07 | 0.002 |
| AGCTC | 5 | 2 | 50 | 25.00 | 0.003 | 0.07 | 0.002 |
| ATAGG | 5 | 2 | 60 | 30.00 | 0.003 | 0.09 | 0.002 |
| ATCTC | 5 | 2 | 60 | 30.00 | 0.003 | 0.09 | 0.002 |
| CCGCG | 5 | 2 | 50 | 25.00 | 0.003 | 0.07 | 0.002 |
| AAAACT | 6 | 2 | 60 | 30.00 | 0.003 | 0.09 | 0.002 |
| AAACGC | 6 | 2 | 204 | 102.00 | 0.003 | 0.29 | 0.002 |
| AAAGCC | 6 | 2 | 90 | 45.00 | 0.003 | 0.13 | 0.002 |
| AAAGGG | 6 | 2 | 120 | 60.00 | 0.003 | 0.17 | 0.002 |
| AAAGTC | 6 | 2 | 330 | 165.00 | 0.003 | 0.48 | 0.002 |
| AAATAC | 6 | 2 | 66 | 33.00 | 0.003 | 0.10 | 0.002 |
| AAATTC | 6 | 2 | 120 | 60.00 | 0.003 | 0.17 | 0.002 |
| AAATTG | 6 | 2 | 90 | 45.00 | 0.003 | 0.13 | 0.002 |
| AACCAC | 6 | 2 | 168 | 84.00 | 0.003 | 0.24 | 0.002 |
| AACCAT | 6 | 2 | 72 | 36.00 | 0.003 | 0.10 | 0.002 |
| AACCGC | 6 | 2 | 162 | 81.00 | 0.003 | 0.23 | 0.002 |
| AACCGG | 6 | 2 | 66 | 33.00 | 0.003 | 0.10 | 0.002 |
| AAGAGG | 6 | 2 | 162 | 81.00 | 0.003 | 0.23 | 0.002 |
| AAGGGC | 6 | 2 | 162 | 81.00 | 0.003 | 0.23 | 0.002 |
| AAGTGC | 6 | 2 | 66 | 33.00 | 0.003 | 0.10 | 0.002 |
| AATATG | 6 | 2 | 84 | 42.00 | 0.003 | 0.12 | 0.002 |
| AATCGC | 6 | 2 | 102 | 51.00 | 0.003 | 0.15 | 0.002 |
| AATGGG | 6 | 2 | 78 | 39.00 | 0.003 | 0.11 | 0.002 |
| AATGTG | 6 | 2 | 84 | 42.00 | 0.003 | 0.12 | 0.002 |
| ACACTC | 6 | 2 | 186 | 93.00 | 0.003 | 0.27 | 0.002 |
| ACACTG | 6 | 2 | 144 | 72.00 | 0.003 | 0.21 | 0.002 |
| ACAGCC | 6 | 2 | 276 | 138.00 | 0.003 | 0.40 | 0.002 |
| ACCTCG | 6 | 2 | 72 | 36.00 | 0.003 | 0.10 | 0.002 |
| ACGGCC | 6 | 2 | 108 | 54.00 | 0.003 | 0.16 | 0.002 |
| ACGGCG | 6 | 2 | 60 | 30.00 | 0.003 | 0.09 | 0.002 |
| ACTCCG | 6 | 2 | 96 | 48.00 | 0.003 | 0.14 | 0.002 |
| ACTCCT | 6 | 2 | 60 | 30.00 | 0.003 | 0.09 | 0.002 |
| ACTGCT | 6 | 2 | 96 | 48.00 | 0.003 | 0.14 | 0.002 |
| AGCCTC | 6 | 2 | 216 | 108.00 | 0.003 | 0.31 | 0.002 |
| ATAGAC | 6 | 2 | 96 | 48.00 | 0.003 | 0.14 | 0.002 |
| ATAGAG | 6 | 2 | 174 | 87.00 | 0.003 | 0.25 | 0.002 |
| ATCACG | 6 | 2 | 174 | 87.00 | 0.003 | 0.25 | 0.002 |
| AACAC | 5 | 1 | 25 | 25.00 | 0.002 | 0.04 | 0.001 |
| AACGG | 5 | 1 | 25 | 25.00 | 0.002 | 0.04 | 0.001 |
| AAGAC | 5 | 1 | 25 | 25.00 | 0.002 | 0.04 | 0.001 |
| ACAGT | 5 | 1 | 25 | 25.00 | 0.002 | 0.04 | 0.001 |
| ACCGC | 5 | 1 | 25 | 25.00 | 0.002 | 0.04 | 0.001 |
| ACCTC | 5 | 1 | 45 | 45.00 | 0.002 | 0.07 | 0.001 |
| ACCTG | 5 | 1 | 25 | 25.00 | 0.002 | 0.04 | 0.001 |
| ACGCT | 5 | 1 | 25 | 25.00 | 0.002 | 0.04 | 0.001 |
| ACTAG | 5 | 1 | 35 | 35.00 | 0.001 | 0.05 | 0.001 |
| ACTGG | 5 | 1 | 25 | 25.00 | 0.002 | 0.04 | 0.001 |
| AGAGG | 5 | 1 | 25 | 25.00 | 0.002 | 0.04 | 0.001 |
| AGCGC | 5 | 1 | 25 | 25.00 | 0.002 | 0.04 | 0.001 |
| AGGCG | 5 | 1 | 60 | 60.00 | 0.002 | 0.09 | 0.001 |
| ATACT | 5 | 1 | 25 | 25.00 | 0.002 | 0.04 | 0.001 |
| ATAGC | 5 | 1 | 25 | 25.00 | 0.002 | 0.04 | 0.001 |
| ATATC | 5 | 1 | 25 | 25.00 | 0.002 | 0.04 | 0.001 |
| ATCCC | 5 | 1 | 35 | 35.00 | 0.001 | 0.05 | 0.001 |
| ATCCG | 5 | 1 | 25 | 25.00 | 0.002 | 0.04 | 0.001 |
| ATCGC | 5 | 1 | 70 | 70.00 | 0.001 | 0.10 | 0.001 |
| ATGAC | 5 | 1 | 35 | 35.00 | 0.001 | 0.05 | 0.001 |
| ATGCC | 5 | 1 | 30 | 30.00 | 0.001 | 0.04 | 0.001 |
| AAAATG | 6 | 1 | 60 | 60.00 | 0.002 | 0.09 | 0.001 |
| AAAGGC | 6 | 1 | 144 | 144.00 | 0.001 | 0.21 | 0.001 |
| AAATCT | 6 | 1 | 30 | 30.00 | 0.001 | 0.04 | 0.001 |
| AAATGT | 6 | 1 | 30 | 30.00 | 0.001 | 0.04 | 0.001 |
| AAATTT | 6 | 1 | 42 | 42.00 | 0.001 | 0.06 | 0.001 |
| AACACC | 6 | 1 | 72 | 72.00 | 0.001 | 0.10 | 0.001 |
| AACAGC | 6 | 1 | 42 | 42.00 | 0.001 | 0.06 | 0.001 |
| AACATG | 6 | 1 | 36 | 36.00 | 0.001 | 0.05 | 0.001 |
| AACTTC | 6 | 1 | 30 | 30.00 | 0.001 | 0.04 | 0.001 |
| AAGAGT | 6 | 1 | 30 | 30.00 | 0.001 | 0.04 | 0.001 |
| AAGCAT | 6 | 1 | 60 | 60.00 | 0.002 | 0.09 | 0.001 |
| AAGGAG | 6 | 1 | 66 | 66.00 | 0.002 | 0.10 | 0.001 |
| AAGGTC | 6 | 1 | 30 | 30.00 | 0.001 | 0.04 | 0.001 |
| AAGTCG | 6 | 1 | 54 | 54.00 | 0.001 | 0.08 | 0.001 |
| AATAGC | 6 | 1 | 30 | 30.00 | 0.001 | 0.04 | 0.001 |
| AATAGT | 6 | 1 | 30 | 30.00 | 0.001 | 0.04 | 0.001 |
| AATCAC | 6 | 1 | 78 | 78.00 | 0.001 | 0.11 | 0.001 |
| AATCCC | 6 | 1 | 48 | 48.00 | 0.001 | 0.07 | 0.001 |
| AATCGT | 6 | 1 | 36 | 36.00 | 0.001 | 0.05 | 0.001 |
| AATGCT | 6 | 1 | 90 | 90.00 | 0.001 | 0.13 | 0.001 |
| AATTAC | 6 | 1 | 30 | 30.00 | 0.001 | 0.04 | 0.001 |
| AATTCC | 6 | 1 | 90 | 90.00 | 0.001 | 0.13 | 0.001 |
| ACACCC | 6 | 1 | 96 | 96.00 | 0.001 | 0.14 | 0.001 |
| ACACCG | 6 | 1 | 54 | 54.00 | 0.001 | 0.08 | 0.001 |
| ACAGGG | 6 | 1 | 48 | 48.00 | 0.001 | 0.07 | 0.001 |
| ACCAGC | 6 | 1 | 48 | 48.00 | 0.001 | 0.07 | 0.001 |
| ACCGGC | 6 | 1 | 30 | 30.00 | 0.001 | 0.04 | 0.001 |
| ACGCAG | 6 | 1 | 42 | 42.00 | 0.001 | 0.06 | 0.001 |
| ACGCTC | 6 | 1 | 84 | 84.00 | 0.001 | 0.12 | 0.001 |
| ACGGAG | 6 | 1 | 102 | 102.00 | 0.001 | 0.15 | 0.001 |
| ACGGGG | 6 | 1 | 60 | 60.00 | 0.002 | 0.09 | 0.001 |
| ACTCAG | 6 | 1 | 108 | 108.00 | 0.001 | 0.16 | 0.001 |
| ACTGGG | 6 | 1 | 36 | 36.00 | 0.001 | 0.05 | 0.001 |
| ACTGGT | 6 | 1 | 60 | 60.00 | 0.002 | 0.09 | 0.001 |
| AGCGGG | 6 | 1 | 60 | 60.00 | 0.002 | 0.09 | 0.001 |
| ATAGCC | 6 | 1 | 30 | 30.00 | 0.001 | 0.04 | 0.001 |
| ATAGGG | 6 | 1 | 36 | 36.00 | 0.001 | 0.05 | 0.001 |
| ATAGTG | 6 | 1 | 60 | 60.00 | 0.002 | 0.09 | 0.001 |
| ATATCG | 6 | 1 | 36 | 36.00 | 0.001 | 0.05 | 0.001 |
| ATCACC | 6 | 1 | 54 | 54.00 | 0.001 | 0.08 | 0.001 |
| ATCATG | 6 | 1 | 30 | 30.00 | 0.001 | 0.04 | 0.001 |
| ATCCCC | 6 | 1 | 42 | 42.00 | 0.001 | 0.06 | 0.001 |
| ATCCTC | 6 | 1 | 66 | 66.00 | 0.002 | 0.10 | 0.001 |
| ATCTCC | 6 | 1 | 138 | 138.00 | 0.001 | 0.20 | 0.001 |
| ATCTCG | 6 | 1 | 30 | 30.00 | 0.001 | 0.04 | 0.001 |
| ATCTGC | 6 | 1 | 42 | 42.00 | 0.001 | 0.06 | 0.001 |

- **Table S11.** Cross-species amplification in *Cephalcia yanqingensis*, *C. chuxiongica* and *C. infumata*.

| Locus | *Cephalcia yanqingensis* | *Cephalcia chuxiongica* | *Cephalcia infumata* |
| --- | --- | --- | --- |
| S1 | 4 | 4 | 0 |
| S2 | 4 | 0 | 0 |
| S4 | 0 | 0 | 0 |
| S5 | 0 | 0 | 0 |
| S10 | 4 | 4 | 1 |
| S11 | 4 | 3 | 0 |
| S16 | 4 | 4 | 1 |
| S18 | 0 | 4 | 0 |
| S20 | 0 | 0 | 0 |
| S21 | 0 | 4 | 0 |
| S24 | 0 | 0 | 0 |
| S25 | 4 | 4 | 0 |
| S27 | 0 | 0 | 0 |
| S28 | 4 | 0 | 0 |
| S29 | 0 | 0 | 0 |
| S30 | 0 | 0 | 0 |
| S36 | 4 | 1 | 0 |
| S39 | 0 | 0 | 0 |
| S41 | 0 | 0 | 0 |
| S42 | 0 | 0 | 0 |
| S47 | 0 | 0 | 0 |
| S48 | 0 | 4 | 1 |
| S50 | 0 | 0 | 0 |
| S55 | 0 | 0 | 0 |
| S62 | 0 | 0 | 0 |
| S63 | 4 | 4 | 4 |
| S64 | 0 | 0 | 0 |
| S74 | 3 | 0 | 0 |
| S76 | 4 | 4 | 4 |
| S85 | 4 | 4 | 1 |
| S90 | 0 | 0 | 0 |
| S92 | 0 | 0 | 0 |
| S94 | 0 | 0 | 0 |
| S98 | 4 | 4 | 1 |
| S99 | 4 | 4 | 0 |
| S100 | 4 | 4 | 0 |
| S103 | 4 | 4 | 1 |
| S107 | 4 | 2 | 0 |
| S110 | 0 | 0 | 0 |
| S111 | 0 | 0 | 0 |
| S113 | 4 | 1 | 4 |
| S114 | 4 | 4 | 1 |
| S116 | 4 | 4 | 1 |
| S117 | 4 | 4 | 0 |
| S125 | 0 | 0 | 0 |
| S129 | 0 | 0 | 0 |
| S132 | 0 | 0 | 0 |
| S133 | 4 | 0 | 0 |
| S134 | 0 | 0 | 0 |
| S135 | 0 | 0 | 0 |
| S141 | 0 | 0 | 0 |
| S144 | 0 | 3 | 0 |
| S148 | 0 | 0 | 0 |
| S149 | 0 | 0 | 0 |
| S150 | 4 | 0 | 0 |
| S159 | 4 | 4 | 0 |
| S160 | 0 | 0 | 0 |
| S162 | 0 | 0 | 0 |
| S164 | 0 | 0 | 0 |
| S176 | 0 | 0 | 0 |
| S185 | 0 | 0 | 0 |
| S188 | 4 | 0 | 0 |
| S190 | 4 | 3 | 0 |
| S202 | 4 | 3 | 0 |
| S205 | 4 | 4 | 4 |
| S206 | 0 | 0 | 0 |
| S207 | 0 | 0 | 0 |
| S213 | 0 | 0 | 0 |
| S214 | 4 | 0 | 0 |

Note: The figure in the table represents the number of individuals successfully amplified, with each species containing 4 individuals; the grey shading implies that all individuals of this species were successfully amplified.

**FIGURES**


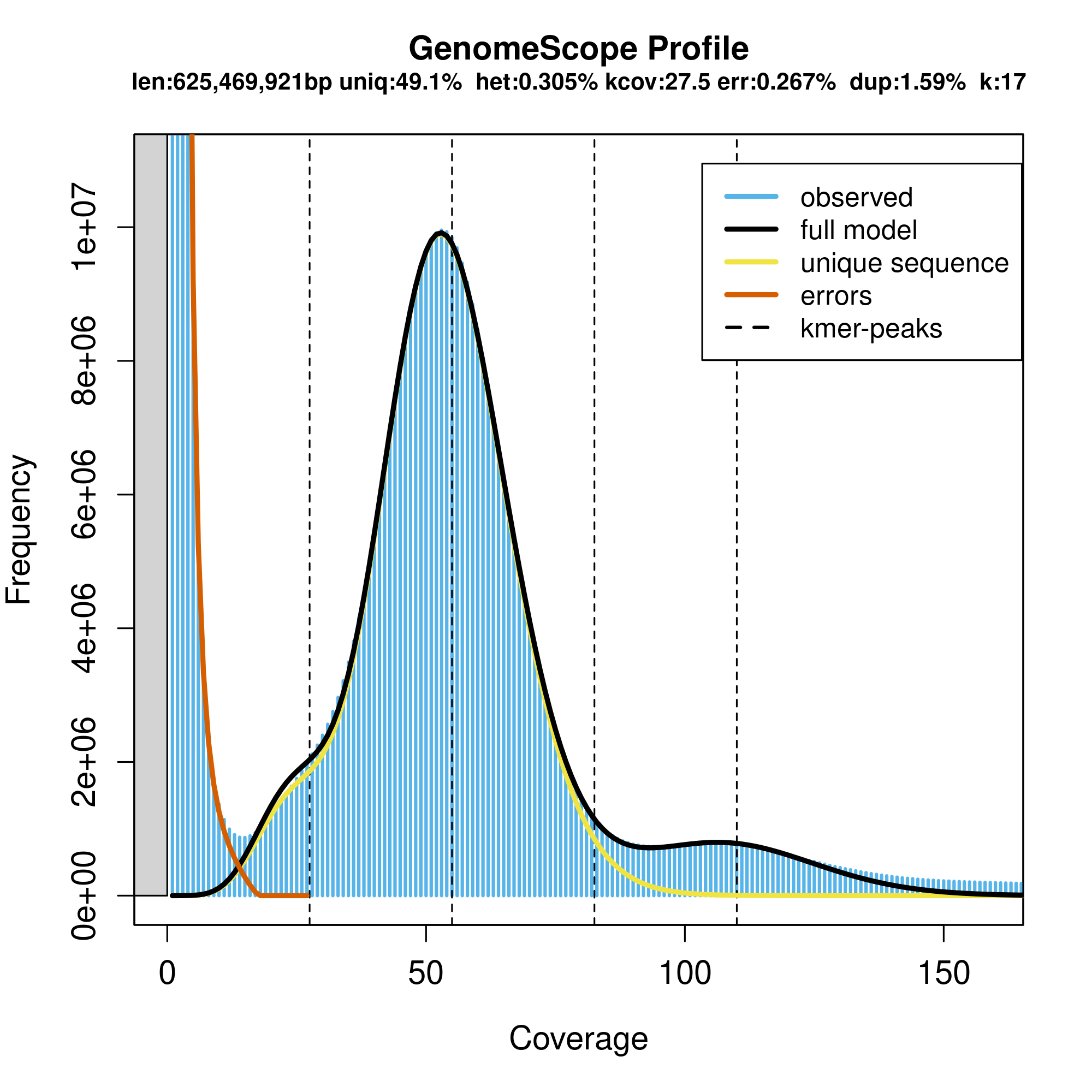


**Figure S1.** GenomeScope analysis of genome size, heterozygosity and duplicate rate using k-mers (K=17) count histogram, indicating a genome size of 625.47 Mb, a heterozygosity of 0.305%, and a duplication rate of 1.59%.


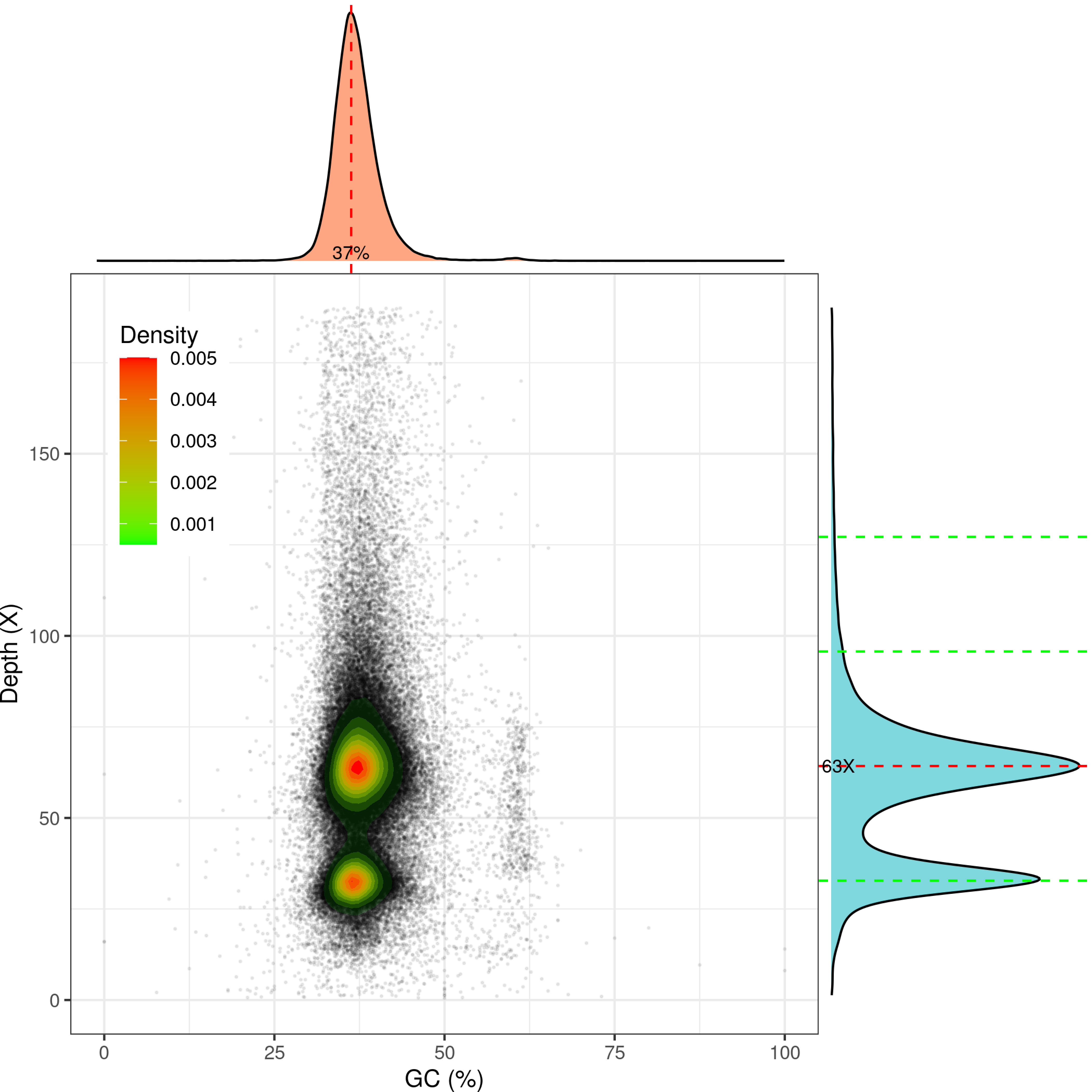


**Figure S2.** GC content and average sequencing depth distribution.


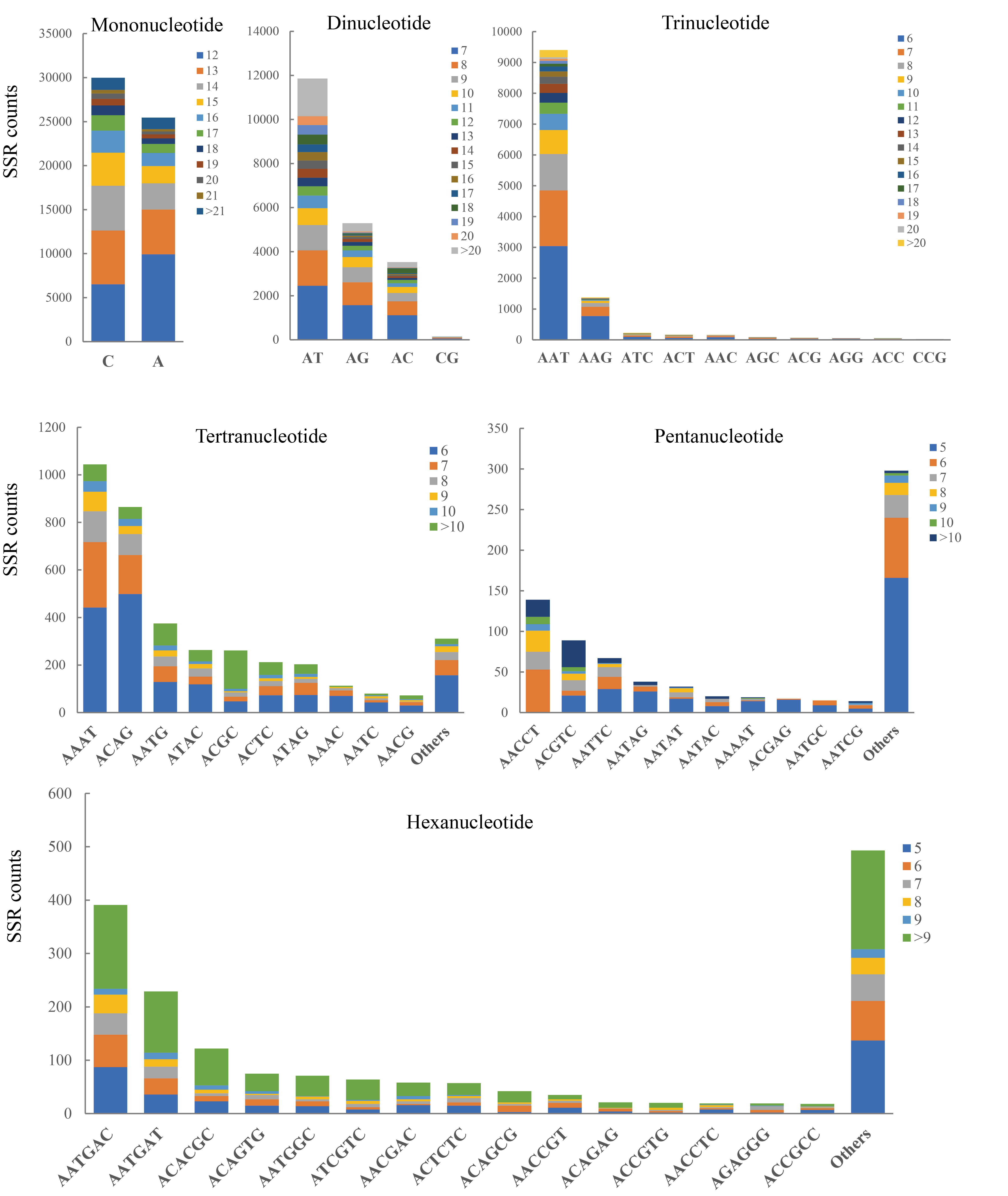


**Figure S3.** Distribution pattern of microsatellite motif types in genome of *Acantholyda posticalis.*
